# Circadian Clock Deregulation and Metabolic Reprogramming: A System Biology Approach to Tissue-Specific Redox Signaling and Disease Development

**DOI:** 10.3390/ijms26136267

**Published:** 2025-06-28

**Authors:** Rossitza Konakchieva, Mitko Mladenov, Marina Konaktchieva, Iliyana Sazdova, Hristo Gagov, Georgi Nikolaev

**Affiliations:** 1 Department of Cell and Developmental Biology, Faculty of Biology, Sofia University “St. Kliment Ohridski”, 1164 Sofia, Bulgaria; r.konakchieva@biofac.uni-sofia.bg; 2 Institute of Biology, Faculty of Natural Sciences and Mathematics, Ss. Cyril and Methodius University, 1000 Skopje, North Macedonia; mitkom@pmf.ukim.mk; 3 Department of Fundamental and Applied Physiology, Russian States Medical University, 117997 Moscow, Russia; 4 Gastroenterology Department, Military Medical Academy, Sofia Center, Ul. “Sveti Georgi Sofiyski” 3, 1606 Sofia, Bulgaria; marina.konaktchieva@yahoo.com; 5 Department of Animal and Human Physiology, Faculty of Biology, Sofia University “St. Kliment Ohridski”, 1164 Sofia, Bulgaria; i.sazdova@biofac.uni-sofia.bg (I.S.); hgagov@uni-sofia.bg (H.G.)

**Keywords:** circadian system, metabolic reprogramming, liver, adipose tissue, proteostasis, redox homeostasis, inflammasome activation, chrono-epigenetics

## Abstract

Circadian rhythms govern cellular metabolism, redox balance, and endocrine signaling in numerous tissues. However, chronic disturbance of these biological rhythms, mediated by modern lifestyle factors including shift work, sleep irregularity, and prolonged light exposure, has been increasingly associated with oxidative stress, metabolic dysregulation, and the pathogenesis of chronic diseases. This review discusses recent mechanistic advances that link circadian misalignment with tissue-specific metabolic reprogramming and impaired proteostasis, focusing on metabolic inflammation and associated pathologies. Emerging work reveals a close interdependence between the circadian clock and proteasome-mediated protein turnover and highlights this interplay’s importance in maintaining redox homeostasis. Furthermore, circadian modulation of the activity of the inflammasome complex is suggested to represent an important, but largely unexplored, risk factor in the pathobiology of both malignancy and metabolic syndrome. Recently, researchers have proposed them as novel endocrine regulators of systemic energy balance and inflammation, with a focus on their circadian regulation. In addition, the emerging domains of chrono-epigenetics and tissue-specific programming of the clock pathways may serve to usher in novel therapies through precision medicine. Moving ahead, circadian-based therapeutic approaches, including time-restricted feeding, chronopharmacology, and metabolic rewiring, have high potential for re-establishing physiological domain homeostasis linked to metabolic inflammation pathologies. Elucidating this reciprocal relationship between circadian biology and cellular stress pathways may one day facilitate the generation of precise interventions aiming to alleviate the health burden associated with circadian disruption.

## 1. Introduction

Circadian rhythms represent endogenous ~24 h cycles driven by molecular clocks and are crucial for physiological homeostasis across most living organisms. In mammals, the central pacemaker residing in the suprachiasmatic nucleus (SCN) of the hypothalamus coordinates peripheral clocks embedded in the liver, adipose tissue, skeletal muscle, and immune cells [1]. These peripheral oscillators tune essential biochemical pathways defined by energy metabolism, immune status, and redox equilibrium by synchronizing internal molecular clocks with external stimuli, including light, feeding, and physical exercise [2]. The circadian molecular clocks at the cellular level drive transcriptional and translational networks controlling proteostasis, mitochondrial dynamics, and redox enzyme function, ultimately promoting metabolic plasticity and adaptation to internal and external challenges.

However, modern life increasingly influences the temporal framework of the circadian timing system. Lifestyle habits, including sleep disruption, nocturnal food intake, and chronic physiological and psychological load, may negatively impact circadian synchrony, resulting in a state of circadian desynchronization [3]. Such desynchronization has been implicated in the pathogenesis of many chronic conditions, including obesity, type 2 diabetes, cardiovascular disease, and immune dysfunction [4,5]. At the molecular level, circadian rhythm misalignment disrupts redox homeostasis through diminished antioxidant defense mechanisms, leading to increased oxidative stress (OS). Mitochondrial dysfunction, defective autophagy, and persistent low-grade inflammation, which are considered hallmarks of metabolic inflexibility and aging-related pathologies [6], are also fostered by redox imbalance.

Recent breakthroughs in chronobiology have uncovered an intricate interplay between the molecular clocks and systemic physiology, including nutrient sensing, circadian hormone secretion, and immune cell migration [7]. Significant advances have been made over the past couple of decades, but important questions remain unresolved, and the most challenging among these is how tissue-specific circadian clocks synchronize with each other and with central metabolic, redox, and immune programs to coordinate whole-body responses to physiological stressors. For example, although the hepatic clock orchestrates gluconeogenesis and lipid metabolism, it is also modulated by inflammatory cytokines and redox-sensitive transcription factors, underscoring the bidirectional integration of these regulatory pathways [8]. Similarly, circadian rhythms in macrophages regulate inflammasome activation and cytokine production, connecting immune surveillance and molecular timekeeping [9].

New evidence emphasizes that, at a systemic level, integrating circadian regulation across different biological systems is essential. The organ-specific interplay among circadian clocks, proteostasis networks, redox regulators, and inflammasome signaling requires detailed characterization [10]. Understanding how circadian misalignment at the cellular level (e.g., aberrant mitochondrial turnover in skeletal muscle) propagates to affect immune function or hepatic metabolism will be key to uncovering the systemic consequences of chronodisruption [11]. Furthermore, identifying critical molecular nodes where circadian control converges with metabolic and immune signaling pathways may reveal novel therapeutic targets directed at restoring homeostatic resilience.

This review aims to put forth a unified chronobiological framework whereby the balance of circadian regulation of proteostasis, redox homeostasis, and inflammasome activation will play a key role. By integrating recent advances in the fields of molecular chronobiology, metabolic inflammation, and redox biology, we seek to highlight existing knowledge gaps and emerging paradigms. Therefore, this integrative view might guide novel chronotherapeutic strategies aimed at improving health quality in the setting of circadian disturbance.

## 2. Circadian Rhythms and Neuroendocrine Adaptation to Environmental Challenges

### 2.1. Circadian Organization as an Adaptive Feature of Mammalian Physiology

The concept of homeostasis as the maintenance of a constant internal environment is a fundamental principle in physiological science, and modern scientific evidence indicates that this constancy is very dynamic. It is a common phenomenon that behavioral functions, both in humans and in other mammals, manifest themselves with pronounced cyclicity, with regular variations during the day (activity/rest cycle), weeks (estrus/menstrual cycle), or months (hibernation, migration). The stability of these rhythms in individuals after removal of external time signals is perceived as evidence for the presence of spontaneous, internal, time-measuring mechanisms, or “biological clocks”. It is believed that the appearance of these rhythms in evolution is associated with the need for synchronization and adaptation of the organism to its characteristic external environment, which can change significantly over time but nevertheless predictably. The manifestation of rhythms can be perceived as an internal sense of time, which gives the individual the opportunity to be in sync with external conditions and thus allows him to optimize his adaptation. Modern analytical and molecular biological techniques have made it possible to demonstrate that the manifestation of overt behavioral rhythms is a synthesis of the superposition of clock-driven physiological states of the internal environment [1,12].

### 2.2. Cyclic Rhythmicity of Neuroendocrine Secretions

The basis of all daily rhythms is the cyclical change in sleep/wake states to meet the challenges and use the opportunities offered by the 24 h periodicity in the shift in light and darkness. The physiological expression of these cycles is reflected in fluctuations of body temperature-metabolic exchange, as well as in neuronal and endocrine activity, which manifest themselves with regular oscillations [13,14]. Important for the endogenous characteristic of rhythms is that when external time signals from the environment are removed, the rhythms continue to manifest themselves with a periodicity of approximately 24 h and are therefore called circadian.

**Figure 1:**
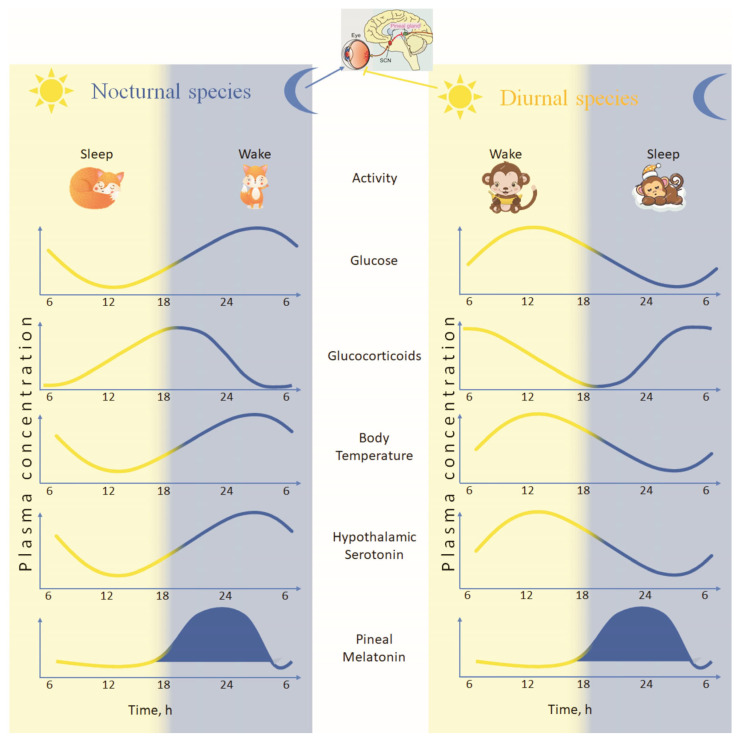
Differences in the temporal organization of circadian rhythms in mammals.

Effective adaptation to changes in the light/dark cycle requires the maintenance of a precise temporal order of neuroendocrine activity and behavior. This is achieved through the interaction of processes with a different circadian profile, both neurobiological and endocrine. For example, for some hormones like glucocorticoids (GCs), the physiological significance of the circadian rhythm of activity has been proven, which is determined by a specific set of target tissues and mechanisms of action [15,16]. The modern view in chronobiological science is that the circadian pattern of secretion is of critical importance for the temporal organization of functions in the body. As in the case of the pineal hormone melatonin, this concerns the circadian and photoperiodic organization of endocrine, behavioral, and metabolic processes (Figure 1). Thus, some endocrine secretions, in particular GCs and melatonin, may have the action of internal synchronizers or “zeitgebers”, responsible for maintaining the temporal order of physiological processes.

### 2.3. Circadian Clocks and Their Importance to Mammalian Homeostasis

The rhythmic nature of various physiological processes, including inflammation and metabolism, is regulated by the endogenous circadian clock, which synchronizes many aspects of human behavior and physiology to changes in the surrounding environment. Light is the main synchronizer for both endocrine and behavioral rhythms, and it has been well established that the master oscillator controlling these rhythms resides in the SCN of the hypothalamus, receiving direct afferents from the retina [13,17].

**Figure 2:**
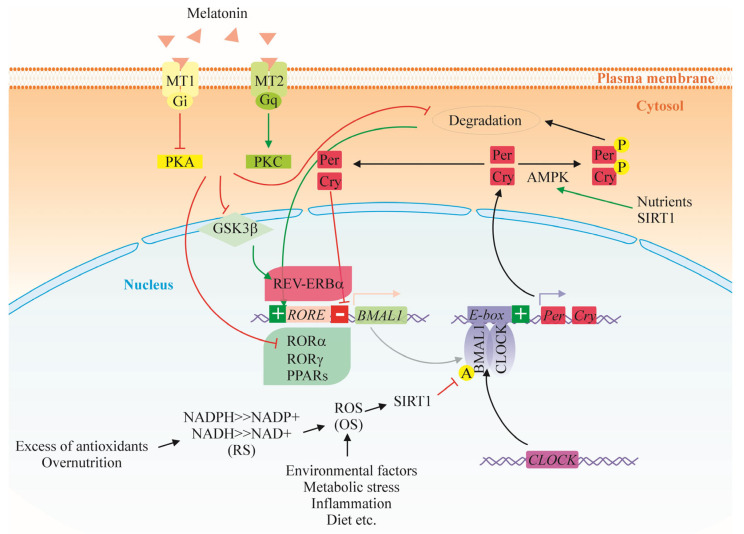
Cellular mechanisms of circadian clocks. CLOCK protein acetylates BMAL1, forming a heterodimer that regulates *Per* and *Cry* expression by E-box promoter. In turn, Per and Cry inhibit *BMAL1* expression, maintaining balance between activators and inhibitors of circadian clocks at the molecular level. High caloric intake increases AMPK activity, phosphorylating Per and Cry, which directs them to degradation. Lower concentrations of Per and Cry activate the expression of *BMAL1*. Redox stress increases SIRT1 deacetylase activity, which inhibits BMAL1/CLOCK dimerization and subsequently reduces the expression of *Per* and *Cry* genes. *BMAL1* expression is augmented by RORα, RORγ, and Peroxisome Proliferator-Activated Receptor (PPARs) or attenuated by REV-ERBβ. Melatonin inhibits proteasomal degradation and RORα as a transcriptional regulator of the *BMAL1* gene. Green lines/boxes (→) indicate activation, red lines/boxes (┬)—inhibition.

Melatonin secretion from the pineal gland acts both as a SCN “master clock output” and “internal time-giver” being tightly controlled by the light-dark cycle [18]. The hormone has been shown to entrain the circadian timing system in experimental rodents via specific membrane receptors in SCN, but little is understood about the relevance of this effect to human physiology except for established circadian rhythm-related sleep disorders, jet lag and shift work- associated disturbances in rest-activity cycle [19]. Sensitivity of the SCN to melatonin in rodents has been demonstrated to occur predominantly during the day-to-night (dusk) and night-to-day (dawn) transition [20,21]. The phase advance of neural activity rhythms in SCN were shown to occur via melatonin type-2 (MT2) receptor activated protein kinase C (PKC) and positive regulation of core clock genes like *Period 1 (Per1)* and *Period 2 (Per2)* [22]. Thus, molecular circadian clock in the SCN may respond to a nonphotic “zeitgeber” like melatonin, which suggests that clock gene transcripts may be targets for clock resetting by internal endocrine and metabolic cues (Figure 2).

Outside the brain, “peripheral” circadian clocks are operational in cells of many other tissues in mammals [23]. It has been documented across different species from mice to human, that intrinsic autonomous circadian oscillations of “peripheral” clocks do not necessarily depend on SCN inputs to keep circadian timing in these tissues. Moreover, transcriptional network of clock genes at cellular level does allow for entrainment of circadian clocks by external and internal cues, under the influence of multiple signaling pathways involved in physiology and metabolism.

Internal secretions and/or environmental factors may also act as circadian cues to either entrain or cause alterations in the clock pathways. Circadian rhythmic patterns of secretions of main endocrine axes are blunted with aging due to the exhaustion of sensitivity of endocrine feedback mechanisms and to the weakening impact of factors influencing the creation of time cycles like decreased retina sensitivity to light. Blunted circadian patterns of key internal factors like glucocorticoids may lead to compromised tissue-specific homeostasis, but also to inadequate metabolic and immune responses [24,25,26,27,28]. Disturbed circadian timing due to shift work, delayed clocks, or certain genetic mutations, can lead to circadian rhythm disruptions affecting sleep patterns, alertness, mood, and overall health. Thus, understanding bodily circadian clock function is crucial for optimizing human health, as it provides insight into sleep disorders, metabolic conditions, mental health, and the development of chronotherapies [29].

### 2.4. Core Elements of the Circadian Clock at the Cellular Level

At the cellular level circadian clock is presented by a complex network of genes and proteins that interact in precise ways, forming interconnected feedback loops [12,30,31]. These loops include transcription factors that activate or repress the expression of clock genes, which in turn produce clock proteins. The clock proteins then modulate the activity of the transcription factors, creating a self-sustaining oscillation with a period of approximately 24 h. One of the key transcriptional activators in the mammalian circadian clock is a heterodimeric protein complex called CLOCK (Circadian Locomotor Output Cycles Kaput)—BMAL1 (Brain and Muscle ARNT-Like 1). CLOCK/BMAL1 controls the expression of other clock genes, encoding the mammalian ortholog *Period* (*Per1*, *Per2* and *Per3*) and CRYPTOCHROME (*CRY1* and *CRY2*) repressor proteins. PER and CRY proteins, in turn, accumulate over time and eventually inhibit their own transcription, forming a negative feedback loop by suppressing the activity of the CLOCK/BMAL1 heterodimer bound at DNA regulatory E-boxes on the target genes. Over time, Per and CRY become phosphorylated and targeted for degradation by AMPK (5′ adenosine monophosphate-activated protein kinase), serine/threonine kinases like casein kinase δ (CK1δ) and CK1, as well as post-transcriptional and post-translational regulation which allows *CLOCK*/*BMAL1* to start a new activation cycle of *Per*/*Cry* gene transcription [1]. AMPK is deeply involved in metabolic homeostasis by acting as a cellular nutrient sensor of low energy states and activator of glucose and fatty acid uptake and oxidation. The stability and cellular localization of the circadian clock proteins are also influenced by other kinases, including GSK3β and various phosphatases [32].

Additional transcriptional feedback loop *CLOCK/BMAL1* activate the genes for inhibitory nuclear receptors *REV-ERBα* and *REV-ERBβ*. Fine tuning of circadian clock functioning is achieved by positive regulation from competitive binding of retinoic acid-related orphan receptors RORα, RORβ, and RORγ on the *BMAL1* gene.

Clock-controlled cell targets include wide array of genes and proteins that are involved in fundamental processes like cell growth and proliferation, apoptosis, metabolism and energy turnover. Among others like SIRT1 (Sirtuin 1) deacetylase, AMPK, GATA1 and NRF2 (Nuclear factor erythroid 2-related factor 2), the tumor suppressor gene *p53* and the oncogene *c-Myc* receive special attention as they implicate the role of circadian control in DNA repair and tumor development [30,33,34,35]

## 3. Chrono-Endocrinology: Limbic Hypothalamic–Pituitary–Adrenal (LHPA)—Pineal Axis and Metabolic Dysfunction

### 3.1. Glucocorticoid Dysregulation and Circadian Metabolic Imbalance

The hypothalamic–pituitary–adrenal (HPA) axis plays a central role in regulating the body’s response to stress, primarily through the circadian secretion of GCs—cortisol in humans and corticosterone in rodents. Cortisol levels correspond to the rest-activity cycle [36] peaking in the early morning, falling in late evening, and reaching a trough during the middle of the night. An abnormally flattened circadian cortisol cycle has been linked with chronic fatigue syndrome, insomnia, and burnout [37]. Limbic and hypothalamic brain structures integrate neuroendocrine, autonomic, emotional, and cognitive stimuli and determine the amplitude and duration of behavioral, neurobiological, and hormonal responses in response to stressful stimuli. Activation of the LHPA axis is the basis of the adaptive response to stress, and adrenal GCs are the main effectors of this system. By influencing gene transcription and the activity of bioelectric potentials of excitable cells, GCs are potent modulators of cellular physiology and behavior. In this way, they are directly responsible for both adaptive and maladaptive processes shaping responses to changing environmental conditions (Table 1). Due to their powerful ubiquitous effects, GCs act as systemic time cues and are instrumental in entraining peripheral clocks and coordinating essential metabolic functions such as gluconeogenesis, lipolysis, and immune modulation [38].

GC signaling is reciprocally regulated by the circadian clock. The core circadian protein BMAL1 regulates the expression of upstream HPA axis components such as corticotropin-releasing hormone (CRH), arginine vasopressin (AVP), and REV-ERBα, the latter of which represses GR-target gene expression in a circadian fashion (Table 1) [39,40].

Chronic stress disrupts this bidirectional relationship by reprogramming clock gene expression and desynchronizing circadian and endocrine rhythms. In rodent models, prolonged stress suppresses corticosterone rhythmicity, dampens hepatic clock gene oscillations, and elevates fasting glucose levels [41,47]. Such chronic stress or circadian misalignment leads to glucocorticoid receptor (GR) desensitization, impairing both circadian signaling and metabolic flexibility, thereby increasing vulnerability to disease [42,48]. Under physiological conditions, GRs regulate transcriptional programs across multiple peripheral tissues, overlapping with core clock components such as BMAL1 and REV-ERBα [44]. Sustained GC elevation—observed in chronic stress, sleep disturbances, or night shift work—can result in GR downregulation, impaired nuclear translocation, and epigenetic repression of GR target genes (Table 1). This desensitization disrupts the temporal coordination of metabolic gene responses, undermining homeostatic feedback during feeding-fasting cycles, and ultimately promoting insulin resistance, adipose tissue inflammation, and hepatic lipid accumulation [43].

Recent findings emphasize the tissue-specific roles of GCs in metabolic regulation (Table 1). In the liver, GR-mediated transcriptional control and chromatin remodeling regulate diurnal expression of key metabolic enzymes, including phosphoenolpyruvate carboxykinase 1 (Pck1), glucose-6-phosphatase (G6pc), and fatty acid synthase (Fasn) [44]. In adipose tissue, GCs modulate local lipolysis and adipokine secretion in a diurnal manner, thereby influencing systemic energy balance. Dysregulated local GC secretion can contribute to metabolic disease, highlighting the importance of peripheral chrono-endocrine dynamics [45].

These insights suggest that targeting the LHPA axis may offer a promising chronotherapeutic strategy for restoring GC rhythmicity and metabolic function (Table 1). Pharmacological approaches, such as selective GR modulators (SGRMs) or timed administration of cortisol synthesis inhibitors (e.g., metyrapone), have shown potential in reestablishing rhythmic GC signaling and ameliorating stress-related metabolic dysfunction [42]. Complementary lifestyle interventions such as TRF, structured physical activity, and bright light exposure may further reinforce endogenous GC rhythms and enhance metabolic resilience.

### 3.2. Circadian Rhythm Desynchronization and Metabolic Disease Predisposition 

The most dramatic example of how the circadian secretory rhythm influences biological functions is illustrated by the secretion of melatonin, known as a “key message of darkness” [49]. In different biological species, this rhythm has a powerful effect on circadian organization—evidence is documented by the use of various approaches such as epiphysectomy, pineal gland transplantation, melatonin treatment, all of which can change the free-running periodicity, the relationship of the cycle phase to light and the overall capacity for the expression of circadian rhythms.

In contrast, the pronounced circadian dynamics in the release of adrenocorticotropic hormone (ACTH) and corticosteroids are generated by modulation of episodic secretion, which results in a more sinusoidal curve describing hormone secretion than that of melatonin. In humans, circulating levels of corticosteroids are highest before awakening and decline progressively as the resting phase approaches, in sync with the decline in the levels of corticotropin-releasing hormone (CRH) and ACTH pulsatile levels. In nocturnal rodents, the peak occurs at the beginning of the active phase and declines during the night. However, in both species (day/night active) the melatonin peak is exclusively during the dark part of the day. As a consequence, the circadian rhythm of melatonin in humans is in antiphase with that of corticosteroids, whereas in nocturnal rodents (rats) the two peaks are parallel. These circumstances imply that the phase ratio is an important criterion for adaptive differences between species, and most likely, different control mechanisms exist for the different rhythms.

Most likely, the overt circadian rhythm is the result of the interaction of two processes: the generation of spontaneous rhythms of hormonal secretion by endogenous internal oscillators, and the synchronization or regulation of oscillators by additional signals (the so-called zeitgebers, synchronizers) [37]. Under normal conditions, these signals synchronize the rhythm generators to oscillate with a periodicity of 24 h and in a certain time order. This leads to the idea that the entire organism can be considered as a system of circadian oscillators, since the generation and manifestation of circadian rhythmicity are interrelated processes.

Melatonin is a known chronobiological agent and in humans, the altered pattern of daily light exposure due to modern lifestyles causes alterations of circadian rhythms characteristics in some individuals. Observed shifts in melatonin diurnal secretion (mesor, peak, amplitude) are probably linked to the established hyperinsulinemia, hypertriglyceridemia, and increased incidence of cardiovascular diseases in these patients, especially in shift workers [50,51,52]. Activity during the dark phase of the day (e.g., in shift workers, modern lifestyles) leads to an impaired insulin response to glucose and is most likely an important risk factor for the development of obesity, night eating syndrome, hypertension, diabetes, and cancer [50,53]. Correlation between melatonin and insulin levels in patients with metabolic syndrome has been shown in a clinical study by Robeva et al. [54] and altered melatonin rhythm was associated with hypercortisolism and immune reactivity shifts in Cushing adenoma patients [25] as well as with hyperinsulinemia and insulin resistance [53].

The notion that melatonin plays an important role in the regulation of endocrine and immune homeostasis, directly or through the activation of effector components of the LHPA axis, and thus able to counteract the consequences of acute stress and associated immune malfunction, is increasingly being recognized. Finally, next-generation omics technologies (e.g., single-cell, single-transcript, and time-series profiling) are essential for mapping tissue-specific GR activity and downstream gene regulatory networks under chronic stress and impaired melatonin pattern of secretion. The development of personalized chrono-endocrine interventions, tailored to individual circadian phase of endocrine secretions, stress exposure, and GR sensitivity, represents a forward-looking avenue for the prevention and treatment of metabolic diseases, including obesity and type 2 diabetes.

## 4. Circadian Regulation of Adipose Tissue Metabolic and Endocrine Function

Peripheral mammalian organs and tissues such as the heart, muscles, liver, and adipose tissues express circadian rhythm activity similarly and short-term independently from the central circadian clock in the hypothalamic SCN [45,55].

### 4.1. Adipose Tissue Diversity and Circadian-Regulated Adipokine Secretion

The adipose tissues (adipose organ) consist of several subcutaneous and visceral depots of adipocytes with adjacent cell types known as a stromal vascular fraction (SVF) of cells, including preadipocytes, fibroblasts, endothelial cells, but also immune cells related to adipose tissue macrophages. The embryonic origin and differentiation status of the adipocytes determines the appearance of several kinds of adipose tissue, comprising the white (WAT) and brown adipose tissue (BAT), as well the beige (brite), pink (breast), and yellow (bone marrow) adipose tissue. Each particular type has a different role in body physiology and disease predisposition [56,57,58,59]. Adipose tissue is now widely recognized as a dynamic endocrine organ that contributes to systemic energy homeostasis, immune regulation, and metabolic control. It participates in a bidirectional crosstalk with the brain and peripheral organs through the release of adipokines—specific signaling molecules of diverse molecular structure, like leptin and adiponectin [60]. Different systemic influences can change the secretion of adipokines and other signal molecules released by SVF and exosome-derived factors [61,62]. Signal-induced changes in local immune reactions can lead to the prevailing of anti-inflammatory or proinflammatory phenotype of the specific adipose tissues with consequences for the metabolism and health performance [45,63,64].

Depot-specific circadian regulation adds complexity to adipose tissue function, as white (WAT), brown (BAT), and visceral fat depots exhibit distinct gene expression patterns and temporal responses to environmental cues. For instance, the circadian clock influences the transcriptional rhythms in murine epididymal white adipose tissue, affecting metabolic processes such as lipogenesis and lipolysis (Table 2). In BAT, circadian mechanisms regulate thermogenic activity, with disruptions leading to increased body fat mass due to attenuated BAT function [65]. Additionally, regulatory T cells in visceral adipose tissue display diurnal variations in activation and metabolism, highlighting the role of circadian rhythms in immune regulation within specific fat depots [66]. These findings underscore the importance of considering depot-specific circadian regulation in understanding adipose tissue physiology and its implications for metabolic health.

In addition to mature adipocytes, adipose-derived stem cells (ASC) demonstrate circadian regulation (Table 2). These multipotent cells hold therapeutic promise due to their ability to differentiate into adipocytes, osteocytes, and chondrocytes [71]. Recent studies show that adipose-derived stem cell (ASC) differentiation, mitochondrial activity, lipid storage, and inflammatory responses are all influenced by intrinsic clocks and external cues. Moreover, ASCs harvested at different times of day show temporal variation in metabolic activity, suggesting that the timing of cell collection could impact therapeutic efficacy [72].

One of the most prominent circadian features of adipose tissue is the rhythmic secretion of adipokines, particularly leptin and adiponectin, which are critical in regulating energy balance and insulin sensitivity. Leptin exhibits a diurnal secretion pattern, with peak levels at night, signaling energy sufficiency to the hypothalamus and promoting energy expenditure (Table 2). In obesity, this rhythm is blunted, leading to leptin resistance and hyperphagia [69]. Similarly, adiponectin, known for its insulin-sensitizing and anti-inflammatory functions, also follows circadian oscillations. Its circulating levels are inversely related to both adiposity and systemic inflammation. Circadian disruption induced by factors such as night shift work, irregular meal timing, or chronic stress can dampen these hormonal rhythms, leading to metabolic inflexibility and increasing the risk of type 2 diabetes [70].

Recent evidence highlights the strong influence of circadian regulation on adipose tissue (Table 2). These rhythms are governed by both central signals from the SCN and intrinsic molecular clocks within adipocytes [68]. These internal clocks, composed of canonical components such as BMAL1, CLOCK, PERs, and CRYs, drive the rhythmic expression of genes involved in lipogenesis, lipolysis, and adipokine secretion [67]. Importantly, external zeitgebers, including feeding patterns, light-dark cycles, GCs, and catecholamines modulate these oscillators [74].

Synchronized expression of circadian oscillator genes *BMAL1*, *Cry1-2*, *Npas2*, and *Per1-3*, and clock-controlled genes *Dbp*, *E4bp4*, *Id2*, *Rev-erb alpha*, *Rev-erb beta*, and *Stra13*, is observed in murine BAT and WAT together with about 650 other genes with circadian expression pattern in WAT, BAT, and liver [67]. Similar results are observed in human subcutaneous adipose-derived stem cells [75] and WAT [76]. Circadian fluctuations are also found in the release of adipokines, which are important for the physiological state of the body as they regulate not only the metabolism of adipose tissues, i.e., the processes of energy storage and expenditure, but also the function of primary homeostatic organs liver and kidney, as well as immune system, muscles, cardiovascular system, and brain [63]. Circadian rhythm desynchronization in adipose tissues as a result of chronic overnutrition, inappropriate diet, or other factors can be a predisposition for obesity and participate as a reason for the related health disorders and diseases [60,63,77].

### 4.2. Circadian Rhythms Disruption as a Precondition for Obesity and Health Disorders

The expression of clock genes in adipose tissues coordinates their metabolism via the activity of nuclear receptors, enzymes, and nutrient transporters of carbohydrates and lipids, as well as the synthesis and release of adipokines [67,78,79,80]. Factors, such as a high-fat diet (HFD), provoke obesity and insulin resistance in animal models, leading to changes in the circadian locomotor activity rhythms due to altered oscillations of clock genes in adipose tissues [81]. Similarly, high dietary fat and carbohydrate content of the diet changes the rhythm of central and peripheral circadian clocks [82]. Therefore, the quality and quantity of nutrients can alter in a metabolism-dependent manner the central and local circadian clocks, i.e., clock gene expression. The reciprocal regulation of metabolic activity by local clocks is also strong. It is observed that the nuclear receptor reverse ERB α (REV-ERBα) down-regulates *BMAL1* expression [83], while retinoic acid receptor-related orphan receptor (ROR) α (RORα) and RORγ [84] and PPARs stimulate *BMAL1* expression via ROR response elements [85]. Other metabolism-related regulators of circadian clocks in adipose tissues are AMPK, an energy status sensor and activator of cellular catabolism, and sirtuins that are nicotinamide adenine dinucleotide (NAD+)-dependent histone deacetylases (Figure 2) [45,63,86] with antioxidant, anti-inflammatory, antiaging and anti-cancer effects [87,88,89].

Behavior and environmental factors like sleep, physical exercise, time and quality of feeding and quantity of food, light/dark alternation, as well as endocrine dysfunction and aging, can either synchronize or disrupt peripheral circadian rhythms in adipose tissues [90]. Overnutrition in middle age leads to metabolic syndrome, which affects the liver, skeletal muscles, pancreas, and heart, which is often preceded by ectopic lipid storage, a process induced when the lipid storage capacity of the adipose organ is overloaded [91,92]. This process often leads to fatty acid derivatives-induced dysfunction followed by lipoapoptosis [92]. Studies on *CLOCK*− and *BMAL1−/−* circadian mutant mice reveal that impaired activity of the self-sustained circadian clock of white adipocytes correlates with lost diurnal lipolytic activity, lower lipid mobilization, declined release of free fatty acids (FFAs) and glycerol into the blood, as well as with enhanced accumulation of TGs in WAT [92]. However, the timing of FFAs release from adipose stores has to be tightly controlled, as an excess of circulating lipids may lead to lipotoxicity and promote cardiovascular disorders. Melatonin can antagonize FFAs lipotoxicity because it regulates circadian gene expression of lipolysis proteins in rat mesenteric WAT and thus prevents elevation of plasma FFAs in HFD [93]. These data support the existence of a strong link between circadian clock disruption and obesity. Moreover, the mutant animals had increased adiposity and high overall body weight [92].

Metabolic crosslink of central and local circadian rhythms dysregulation and OS, and most of their mechanisms are well known, but it is still not fully understood which of them is causative for the initiation of pathological processes, i.e., has the primacy in diet and obesity-related dysfunctions. To clarify this subject, the mechanisms of behavior and metabolic factors that induce circadian clocks desynchronization can be compared to estimate the time when reactive oxygen species (ROS) imbalance appears. The expression of common adipocytokines in rodents and humans shows strict circadian rhythmicity, while dietary fat and carbohydrates alter the function of the central and peripheral circadian clocks [82]. In male Wistar rats, diurnal sympathetic activity, but not regular feeding, and insulin or corticosterone signals, is essential for the regulation of the daily rhythm of adipocyte leptin release and its plasma level [94]. Therefore, it can be suggested that the central and peripheral circadian clocks’ desynchronization precedes OS. This opinion is supported by the observation of quick disruption of the human circadian clock due to transition to daylight saving time in spring, a shift that increases health risks [95] and is related to insufficient sleep, lower cognitive performance, poorer mental health, and an increased risk of motor vehicle accidents [96,97]. Additionally, in overnutrition and the related obesity, OS is preceded and, to some extent, provoked by reductive stress [87,98]. When generated, chronic OS deepens central and local circadian clocks dysfunction, i.e., serves as a positive feedback regulator for the generation of pro-pathogenic conditions [99,100,101,102].

Adipose tissue innervation, particularly through the sympathetic nervous system (SNS), is also rhythmically regulated. The SNS promotes lipolysis in WAT and BAT, following a diurnal pattern under SCN control (Table 2). Disruption of this SNS-adipose axis impairs metabolic flexibility and thermogenic capacity, contributing to the progression of obesity and related metabolic disorders [73].

Moving forward, systematic profiling of circadian transcriptomes in adipose depots under both physiological and pathological conditions will be essential to identify clock-controlled genes involved in adipokine secretion, endocrine signaling, and metabolic adaptation. Additionally, time-based therapeutic interventions such as TRF, chronopharmacological targeting of adipose clocks, and circadian-timed ASC transplantation hold substantial potential to improve metabolic health in conditions like obesity and type 2 diabetes.

### 4.3. Oxidative Stress, Obesity, and Adipose Tissue Secretion

**Figure 3:**
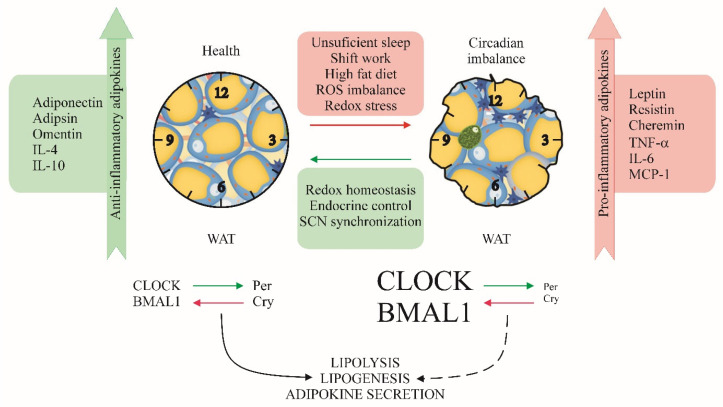
Regulation of circadian clocks in adipose tissues. In healthy state (left side), adipose tissues secrete predominantly anti-inflammatory adipokines, which is supported by appropriate endocrine regulation and redox status. Many factors including diet and sleep disturb the adipose tissue functionality (right side), which increase proinflammatory signaling. Green arrows (→) indicate positive effect, red arrows (→)—negative effect, and dotted line—impaired regulation. Increased font size symbolizes higher concentration, decreased font size—lower concentration.

Fat accumulation in obesity leads to NADPH oxidase-induced OS and dysregulation of adipocytokines’ production. This is manifested with lower plasma levels of adiponectin due to its downregulated transcription, increased lipid peroxidation in nondiabetic subjects, and increased expression of proinflammatory *IL-6*, monocyte chemotactic protein-1 (*MCP-1*), and with less expression of plasminogen activator inhibitor-1 (*PAI-1*) in 3T3-L1 adipocytes [103]. Thus, systemic obesity-related OS, independent of hyperglycemia, increased OS in obesity might relate to the dysregulated production of adipocytokines. A decrease in plasma adiponectin in obesity correlates with body mass index (BMI) and is causative of insulin resistance and atherosclerosis [103]. In diabetic type 2 humans with high BMI, abdominal obesity, and total obesity have a strong influence on plasma levels of adipokines adiponectin, leptin, and resistin, as the last two increased and similarly change the inflammatory markers high-sensitive C-reactive protein (hsCRP), Tumor necrosis factor-α (TNF-α), and IL-6 (Figure 3) [104].

Expression of anti-oxidative enzymes and production of antioxidants express circadian rhythm and this activity is impaired in OS [6,45,105]. In obesity, perivascular adipose tissue induces hypoxia, inflammation, and OS [106]. The hypoxia relates to adipocyte hypertrophy, which reduces angiogenesis and capillary density [107]. Obesity upregulates proinflammatory adipokines and downregulates anti-inflammatory adipokines release from adipose tissues, thereby contributing to the pathogenesis of diseases in the cardiovascular system and other body structures [107]. The changed secretory activity provokes infiltration of immune cells, leading to low-grade inflammation that is further enhanced by cytokines from B and T cells, neutrophils, macrophages, and mast cells, whose number is increased in visceral adipose tissue of obese humans and mice [108].

OS is an imbalance between free radicals and antioxidants, leading to various harmful effects on health. These include inflammatory neutrophil infiltration, increased secretion of proteases, and increased production of oxidative intermediates, all of which significantly contribute to aging and the onset of multiple diseases. A deeper understanding of OS, reductive stress, obesity, circadian lipid metabolism, and adipokine secretion could reveal new therapeutic approaches for the treatment of metabolic disorders.

### 4.4. Kisspeptin as a Link Between Circadian Activity, Reproduction, and Metabolism

The kisspeptin gene *Kiss1* encodes the neuropeptide kisspeptin, initially known as human metastasis suppressor (metastatin), in the hypothalamic anteroventral periventricular nucleus (AVPV) and arcuate nucleus [109,110]. Hypothalamic kisspeptin binds to a G-protein-coupled kisspeptin receptor on GnRH neurons in the median eminence, activates GnRH secretion, and triggers LH and FSH release from the anterior pituitary gland downstream. This hypothalamus-pituitary-gonadal (HPG) axis activation initiates puberty and supports the proper function of the reproductive system, as in females leads to oocyte maturation, and ovulation due to LH and estradiol surge, and in males regulates testosterone synthesis, spermatogenesis, and behavior [111,112]. In this way, kisspeptin is involved in pubertal maturation, supports the gonad function and fertility, and participates in age-related LH dysfunction, idiopathic hypogonadotropic hypogonadism, central precocious puberty, and infertility, as observed in Sprague Dawley rats, other rodents, and humans [111,112,113,114].

Circadian rhythms of LH and *Kiss1* expression in hypothalamic kisspeptin neurons peaked simultaneously, which suggests SCN-dependent regulation of circadian activity of reproductive neurons in the female HPG axis [109,110]. This hypothesis was supported by the expression of BMAL1 and other clock genes in kisspeptin neurons of AVPV of female mice [115], as well as by the circadian rhythm of hypothalamic *Kiss1* expression of female Wistar rats [116]. Additionally, toxin-induced silencing of *Kiss1* in arcuate nucleus neurons of adult female mice shifts food consumption and wakefulness to the light phase, and attenuates temperature rhythms [117]. Under these conditions, the rhythm of the SCN master clock is intact, which suggests that kisspeptin neurons of the arcuate nucleus participate as an important part of the hypothalamic circadian oscillator network.

*Kiss1* is also expressed in peripheral tissues. In 2008, it was found that *Kiss1* expression in adipose tissue was independent of the hypothalamus but was sensitive to sex steroids and nutrition as food restriction for 24 h increased kisspeptin mRNA in adipocytes of Sprague-Dawley rats of both sexes, while a HFD for the same period reduced [118]. Impaired kisspeptin signaling decreases metabolism and promotes glucose intolerance and obesity. Increased adipose tissue mass in obese subjects is responsible for increased expression of kisspeptin and kisspeptin receptors, and for higher plasma levels of kisspeptin [119,120]. As a result, kisspeptin signaling and its role in glucose homeostasis are enhanced in parallel to body weight [119]. Kisspeptin receptor knockout mice of both sexes are characterized by infertility and higher fat mass, while female mice only show lower food intake, locomotion, energy expenditure, and glucose tolerance [121].

In adult male rhesus monkeys, short-term fasting and human kisspeptin 1-10 applied intraperitoneally regulate the release of major adipokine adiponectin [122]. It was observed that the plasma level of adiponectin increased under fed and fasting conditions. The administration of kisspeptin 1-10 additionally increased adiponectin level but left the plasma concentrations of leptin and resistin unchanged [122]. Thus, kisspeptin indirectly via adiponectin may have pleiotropic effects on metabolism by increasing glucose uptake and utilization in health and diabetes, insulin sensitivity, fatty acids oxidation, decreasing fat storage, and reducing inflammation [123]. In obese rodents and humans, kisspeptin secretion from adipose tissue or by glucagon-stimulated release from the liver is important for its plasma accumulation and can dominate over its release as a neuropeptide for GnRH and HPG axis regulation [119,120,121]. Application of kisspeptin 1-10 inhibited cell proliferation, adipogenesis, and viability in mouse 3T3-L1 cells; increased lipolysis in 3T3-L1 cells and rat adipocytes by enhancing the expression of perilipin and hormone-sensitive lipase, and increased secretion of leptin, glucose uptake, and lipogenesis in rat adipocytes [124]. Additionally, central and peripheral expression of the *Kiss1* is under hormonal, metabolic, and environmental control. Kisspeptin neurons are excited by the circadian activity of arginine vasopressin during the LH surge in female mice, as well as relaxin-3 and leptin in mammals, while dynorphin in male rats, ghrelin in rats and sheep, somatostatin in sheep, and substance P in female mice suppress their function [125]. All these data suggest that kisspeptin, as a neuropeptide, a paracrine factor or hormone, is an important regulator of metabolism and fertility, linking circadian rhythm, nutrition, and obesity [125]. Moreover, in addition to the hypothalamus, liver, adipose tissues, and gonads, kisspeptin and its receptor expression are observed in the pituitary gland, pancreas, small intestine, and placenta. Further studies are required to make a picture of the overall systemic physiological regulations of kisspeptin.

Kisspeptin links body mass and sex hormones, especially estradiol and testosterone [126]. In women, low levels of sex steroids after menopause, and in men, decreased plasma testosterone concentration, often lead to higher body mass index [127,128]. The inhibitory effect of estradiol on food intake is well-documented in animals, e.g., rats and mice [129]. The underlying mechanism includes estrogen-dependent activation of the ghrelin receptor expression in kisspeptin neurons in the arcuate nucleus, which reduces *Kiss1* mRNA in rats [130]. Additionally, kisspeptin neurons of the hypothalamic arcuate nucleus could serve as intermediate neurons in the appetite regulation due to direct inputs from agouti-related protein and proopiomelanocortin neurons, as well as vice versa, by kisspeptin and glutamate, whose secretion is stimulated in the presence of estradiol [126].

## 5. Circadian Control of Cellular Proteostasis and Redox Balance

### 5.1. The Proteasome-Circadian Interface in Metabolic Regulation

Cellular proteostasis—the finely regulated balance of protein synthesis, folding, and degradation—underlies the maintenance of metabolic and redox homeostasis. At the center of this system lies the ubiquitin-proteasome system (UPS), an ATP-dependent pathway responsible for the selective degradation of misfolded, damaged, or short-lived regulatory proteins [131]. New findings also demonstrate a collaboration between circadian clocks and proteasomal activity that highlights temporal modulation of protein degradation as a critical function of cellular and systemic homeostasis [132]. Disturbances of this circadian-dependent proteostasis axis have been increasingly linked to the pathogenesis of metabolic disorders, such as obesity, insulin resistance, and type 2 diabetes (Table 3) [133].

At the molecular level, the core circadian transcription factors, BMAL1 and CLOCK, govern rhythmic expression of genes associated with metabolic and redox processes, including components of the UPS [134]. To begin, proteasomal function itself follows circadian oscillations, showing tissue-specific variation in the subunit expression and proteolytic activity over the diurnal cycle [135]. For instance, rhythmic expression of 20S and 26S proteasome subunits in mouse liver and skeletal muscle corresponds with the turnover of major metabolic enzymes and mitochondrial proteins. These rhythms are not just by-products of metabolic activity but are actively regulated by networks of clock-controlled transcriptional and redox-sensitive signaling cascades [132].

BMAL1 is key in coordinating proteasome dynamics. BMAL1 protein deficiency caused by genetic deletion reduced the assembly of the proteasome, decreased the degradation of oxidatively modified proteins, and increased the accumulation of misfolded proteins [24]. BMAL1 deficiency also disrupts proper mitochondrial quality control pathways, inducing the loss of the turnover of proteins regulating mitophagy and mitochondrial biogenesis like Peroxisome proliferator-activated receptor gamma coactivator 1-alpha (PGC-1α) and Nuclear respiratory factor 1 (NRF1), which are also subject to circadian proteasomal degradation [136]. This regulation is critical for the maintenance of mitochondrial function, efficiency of oxidative phosphorylation, and minimization of mitochondrial ROS production during metabolic transitions [24].

In metabolic disease, the circadian regulation of proteostasis becomes severely dysregulated. For example, in models of diet-induced obesity, proteasome subunit expression oscillations are suppressed, leading to compromised protein turnover and elevated oxidative stress and chronic inflammation [139]. In addition, metabolic stressors including hyperglycemia and nutrient excess may dysregulate clock gene expression and function, establishing a feedback loop that decouples proteasomal activity from circadian regulation. Such misalignment aggravates redox imbalance and proteotoxic stress of metabolically active organs, including the liver, pancreas, and skeletal muscle [138].

Recent research highlights the circadian modulation of antioxidant defense mechanisms mediated through proteasomal systems (Table 3). Circadian regulation of gene expression is associated with the circadian clock, which regulates a diverse set of pathways and genes, including the master transcriptional regulator of antioxidant genes *Nrf2*, that is transcriptionally and post-translationally regulated in a circadian manner [35]. The possibility of NRF2 to undergo proteasomal degradation can be induced by a variety of cytotoxic and genotoxic stressors. As a highly conserved process, it is known to be regulated by the circadian protein BMAL1 [140]. Together, these results uncover an indirect but essential connection between circadian clock components and redox homeostasis, mediated by circadian proteasomal regulation of antioxidant defense [140].

There is increasing evidence for the role of melatonin as a chronobiotic regulator of the circadian clock through interaction with the ubiquitin–proteasome system and its impact on SIRT1 and c-Myc. The upregulation of SIRT1 by SRT1720 (SIRT1 activator) attenuates melatonin’s antioxidant and antitumor activity, indicating that its induction of ROS production in tumor cells is activated by SIRT1 [141]. In turn, inhibition of SIRT1 levels by melatonin produces the downregulation of the MDM2 pathway, a ubiquitin protein ligase, which enhances p53 activity through acetylation. It is suggested that melatonin influences SIRT1 through its specific receptor MT1, blocking the transcription of *BMAL1* and therefore SIRT1 via inhibition of RORα (Figure 2) [142].

Taken together, these observations highlight the proteasome-circadian clock interface as a critical regulatory hub for the maintenance of cellular homeostasis. This axis is an attractive target for the treatment of metabolic disease, especially those that are consequences of both disrupted rhythmicity and impaired protein quality control. Such studies should pursue methods to restore rhythmic proteasomal activity, either via pharmacological UPS function enhancers or interventions to boost clock gene expression. In addition, further tissue-specific studies will be needed to clarify how tissue-specific clocks integrate local proteostatic and redox cues into systemic metabolic responses.

### 5.2. Circadian Inflammasome Activation and Metabolic Inflammation

Inflammasomes are complexes of several proteins that serve as intracellular sensors of metabolic stress, infection, and cell damage, which trigger the activation of caspase-1 and the inflammation-potent precursors of inflammatory cytokines, especially IL-1β and IL-18 [143]. Among the characterized inflammasomes, NOD-like receptor family pyrin domain containing 3 (NLRP3) and Absent in Melanoma 2 (AIM2) are central module mediators of innate immune response, particularly about metabolic inflammation (Table 4). Increasing evidence demonstrates that the activity of these effector complexes is not constant but rather exhibits circadian rhythms under the control of conserved molecular clock components such as BMAL1, CLOCK and REV-ERBα (also called NR1D1), nuclear receptor and core component of the circadian clock [9,144]. Nevertheless, the mechanistic basis for the interplay of circadian regulation of the inflammasome, redox signaling, and chronic metabolic disease is not yet elucidated.

An important open mechanistic question is how rhythmic inflammasome activation integrates with pathways related to OS to maintain chronic low-grade inflammation in metabolically active tissues such as the liver, adipose tissue, and the bone marrow. Circadian clocks promote cellular redox homeostasis by temporally regulating the expression of antioxidant enzymes, including peroxiredoxins and glutathione peroxidases [6]. Overt circadian rhythm disruption induced by either night shift work, sleep deprivation, or misaligned feeding renders this redox regulation dysfunctional, leading to mitochondrial impairment, increased ROS generation, and the higher priming and activation of the NLRP3 inflammasome [145]. Mitochondrial ROS also has a prominent role in the proximal inflammatory signal of inflammasome activation, whereas components of the clock, including BMAL1, help to curtail ROS generation by promoting mitochondrial integrity [136].

Studies in various animal models provide compelling evidence that circadian desynchronization amplifies inflammasome signaling in metabolic tissues (Table 4). In addition, *NLRP3* expression and IL-1β production are upregulated in liver and adipose tissue in mice subjected to chronic jet lag or on a HFD during the rest phase [146,147]. These temporally misaligned activations of inflammasomes are responsible for macrophage infiltration and the increased risk of developing insulin resistance and hepatic steatosis and fibrosis, which are well-known features of non-alcoholic steatohepatitis (NASH, new MASH, 2023) [148].

In turn, many in vitro and in vivo studies have proved that melatonin alleviates NLRP3 inflammasome activity via various intracellular signaling pathways. In the context of metabolic disease [150]. Melatonin exerts inhibitory function on NLRP3 inflammasome activation through inhibiting or activating several proteins and pathways. Nuclear factor kappa B (NF-κB) is a master regulator of the priming phase of NLRP3 inflammasome activation. Melatonin prevents NLRP3 inflammasome activation by inhibiting NF-κB signaling via RORα and silent information regulator 1 (SIRT1)-dependent deacetylation of NF-κB [151]. ROS is a main trigger of NLRP3 inflammasome activation. Growing evidence shows that melatonin reduces levels of thioredoxin-interacting protein (TXNIP), leading to suppression of ROS production and NLRP3 activity [152]. In the liver, various deleterious effects of excess NLRP3 activity have also been shown in mouse models but also in patients [153]. Non-alcoholic hepatosteatosis in mice involves constitutively active NLRP3 leading to widespread pyroptosis, fibrosis, and elevated inflammatory marker expression (IL-1β, TNF-α, caspase-1). Daily IP melatonin injections in a cadmium-induced liver injury model decrease IL-1β levels in serum, attenuates hepatocyte death, reduces protein levels of NLRP3 and TXNIP, ROS levels, caspase-1 activity, and TXNIP-NLRP3 interaction in liver tissue of cadmium-administered mice [152].

The circadian transcriptional repressor, nuclear receptor REV-ERBα, has emerged as a key link between temporal regulation and immune-metabolic homeostasis. REV-ERBα pharmacological activation reduces NLRP3 inflammasome activation in macrophages and ameliorates diet-induced metabolic inflammation [149]. Deletion of *BMAL1* in myeloid cells also promotes inflammasome priming and cytokine production, which corroborate that innate immune activation is dampened by endogenous circadian mechanisms [9].

Collectively, these findings are consistent with the proposal that circadian misalignment acts as a major driver of inflammasome-driven inflammation in metabolic disease. Future studies need to address the temporal dynamics of inflammasome activity within specific tissues and immune cell subsets, particularly in human models. Inflammasome oscillatory high-resolution profiling may reveal early biomarkers of metabolic disease evolution and stimulate chrono-immunotherapeutic strategies.

## 6. Tissue-Specific Metabolic Clocks: Beyond the Liver and White Adipose Tissue

### 6.1. Circadian Control of Bone Marrow Adipose Tissue and Pink Adipose Tissue

While the circadian regulation of liver metabolism and white adipose tissue (WAT) has been well defined, the roles of less conventional fat depots, like bone marrow adipose tissue (BMAT) and pink adipose tissue, have been shown to have chronobiological functions but remain largely unexplored [78]. We are still on the frontier of circadian biology concerning these more specialized adipose tissues, as emerging evidence suggests that these tissues have unique metabolic and endocrine functions, which may be subject to circadian modulation and have wide-reaching effects on systemic energy homeostasis, hematopoiesis, and immune regulation (Table 1) [154].

In humans, ≈10% of total adipose mass is comprised by BMAT, which is strategically deposited within the bone marrow cavity and expands in association with aging, caloric restriction, and metabolic disease [155,156]. BMAT, in contrast to classical WAT or thermogenic BAT, is only modestly responsive to β-adrenergic stimulation and has a unique transcriptomic and lipidomic profile. Despite these differences, its temporal regulation and its relationship to circadian clocks are poorly defined. Recent transcriptomic analyses show that BMAT expresses the core clock genes *BMAT1*, *Per2*, and *Rev-erb alpha* with evidence of diurnal variation, showing that they play the role of an intrinsic molecular clock [145]. Taking that bone marrow hematopoiesis and immune cell trafficking are under circadian control [157], it is plausible that BMAT contributes to chronobiological coordination by synchronizing energy storage and mobilization with the rhythmic metabolic and proliferative demands of hematopoietic cells (Table 4).

One central hypothesis is that BMAT mediates immune-metabolic crosstalk via the time-of-day dependent release of lipids, adipokines, and exosomes, subsequently regulating hematopoietic stem cell (HSC) maintenance, lineage commitment, and systemic inflammation. Exosomes secreted by BMAT contain circadian-regulated cargo such as microRNAs and mitochondrial proteins, that respond to both external zeitgebers (e.g., feeding and light cycles) and internal stressors [158,159]. Circadian disruption as a result of night shift work or aging may disturb this exosomal signaling system, thereby promoting dysregulated hematopoiesis, inflammation, and bone demineralization [158]. Certainly, accumulation of BMAT has been associated with dysregulated osteoblast differentiation and enhanced fragility of bones, supporting the hypothesis that circadian maladaptation of BMAT may serve a putative role in specific components of the development of osteoporosis [156,160]. Simultaneously, pink adipose tissue—as a transient thermogenic store observed during lactation in the mammary fat—is qualified as another less-examined domain within the research of circadian rhythms (Table 5). Pink fat, derived from transdifferentiated white adipocytes, displays plasticity in mitochondrial function and in secretory capacity, which could be subject to circadian regulation [161].

Clinically, the circadian regulation of BMAT and pink adipose tissue may have important implications. Both older adults and night shift workers’ populations, frequently affected by circadian misalignment, exhibit increased BMAT, which correlates with higher risk for bone fractures, anemia, and immune dysfunction [162]. Deciphering the temporal dynamics of BMAT may thus uncover novel biomarkers of skeletal and immune aging and open new avenues for chrono-therapeutic interventions aimed at preserving bone and marrow health (Table 5).

Future studies will need to take an integrative, time-resolved approach, utilizing transcriptomics, proteomics, and metabolomics to characterize circadian signals in BMAT and pink adipose tissue. Moreover, revealing exosome-mediated communication between these adipose depots and adjacently residing hematopoietic or epithelial compartments could show how circadian mismatch destabilizes the local tissue integrity along with systemic homeostasis.

### 6.2. Circadian Epigenetic Reprogramming in Liver Metabolic Disorders

The liver is a master organ that plays a central role in nutrient metabolism, detoxification, and hormone processing, which are tightly regulated by the circadian clock [163]. Increasing evidence indicates that time-dependent chromatin remodeling has a crucial role in the regulation of hepatic metabolic processes. In this new frontier of circadian epigenetics, cell-type specific genome-wide changes in histone modifications and chromatin accessibility govern the regulation of genes structuring lipid metabolism, gluconeogenesis, and xenobiotic clearance. Disturbances in these rhythms have been associated with the pathogenesis of chronic liver diseases, such as metabolic dysfunction–associated liver disease (MASL) and metabolic dysfunction–associated steatohepatitis (MASH) in which steatosis is combined with inflammation and sometimes fibrosis (Table 6), a risk factor for complications such as cirrhosis and hepatocellular carcinoma [163,164].

The central clock transcription factor BMAL1 is a chromatin modifier and not only a transcriptional regulator. It binds to circadian enhancers and recruits histone acetyltransferases (HATs) like p300, thereby promoting rhythmic acetylation of histone H3 at lysine 9 (H3K9ac), which is associated with transcriptional activation [164,166]. This circadian acetylation is enriched at promoters of genes controlling lipid biosynthesis (e.g., *Fasn*, *Scd1*, *Acaca*) and detoxification pathways (e.g., *Cyp* family genes) and thereby synchronizes hepatic gene expression with feeding-fasting cycles [167]. Disruption of this rhythmic acetylation, whether by chronic HFD, chronic circadian misalignment, or genetic perturbation of some components of the clock machinery, leads to aberrant metabolism characterized by the accumulation of lipids, disruption of bile acid metabolism, and increased OS (Table 6), [174,175].

Environmental and dietary factors can induce circadian-dependent reprogramming of the hepatic epigenome, with effects that persist long after the initial exposure [170]. For instance, an HFD has been shown to abolish the diurnal rhythms of histone modifications, such as H3K9ac and H3K4me3 [165]. This disruption compromises the timing of transcription, rendering the liver more susceptible to steatosis and inflammation (Table 6). Additionally, circadian disruptions caused by factors like night shift work and irregular feeding schedules have been linked to aberrant rhythmic DNA methylation and chromatin architecture, leading to impaired hepatocyte function and increased liver injury [169]. For instance, a study by Ding et al. (2022) [168] investigated the effects of an HFD on hepatic and adipose circadian rhythms in gestational mice. The findings revealed that the HFD significantly disrupted the rhythmic patterns of circadian clock genes and downstream metabolic genes in the liver, highlighting the impact of dietary factors on circadian regulation and liver metabolism.

These observations indicate that circadian histone markers are valuable therapeutic targets. Chrono-pharmacology approaches based on delivering epigenetic modulators (e.g., histone deacetylase (HDAC) inhibitors at specific time points of the circadian cycle are likely to augment therapeutic efficacy while limiting toxicity in metabolic liver disorders (Table 6). h Th time-dependent effects of HDAC inhibitors, such as valproate and butyrate, on hepatic gene expression and mitochondrial activity have been shown in preclinical studies [171,176]. Also, it has been shown that targeting the NAD^+^-dependent deacetylase (SIRT1) with circadian activity restores clock-regulated chromatin dynamics while ameliorating steatosis in models of human NAFLD [172].

The emerging field of chrono-epigenetics—where circadian and epigenetic regulation intersect—provides a new paradigm for precision medicine. Circadian pathways also provide a new framework for understanding and potentially treating liver diseases. Circadian and epigenetic profiling of individuals could lay the basis for personalized therapy in MAFLD, liver fibrosis, and even hepatocellular carcinoma, potentially guided by oscillatory biomarkers such as circadian H3K9ac or time-specific transcriptomic profiles (Table 6). In addition, lifestyle changes such as TRF, which entrain circadian rhythmicity independent of caloric restriction, can restore histone acetylation rhythmicity and protect against diet-induced hepatic pathology [173].

Future investigations should aim to comprehensively identify circadian epigenetic markers in the liver and delineate their interactions with metabolic cues, inflammatory mediators, and fibrotic pathways. Deciphering these multilayered regulatory networks will be critical for advancing time-based strategies to prevent and treat liver disease.

## 7. Translational and Clinical Implications: Future Research Directions

### 7.1. Chrono-Pharmacology and Metabolic Disease Treatment

The circadian system is responsible for orchestrating the rhythmic activities of metabolic pathways, hormonal secretion, gene expression, and drug metabolism Table 7. These intrinsic biological rhythms can be forced out of alignment, however, leading to impaired metabolism and obesity [177]. Chrono-pharmacology is an emerging discipline that studies the impact of administering a treatment at a specific time in the circadian cycle to promote the therapeutic efficacy and reduce adverse effects. A salient and unanswered question is whether time-optimal pharmacological strategies can improve therapeutic outcomes as well as circadian homeostasis and thus support metabolic health.

The principles of chrono-pharmacology are based on the fact that drug pharmacokinetics and pharmacodynamics are time-dependent (Table 3). An important pharmacological feature, for instance, is the circadian variation in processes such as drug absorption, distribution, metabolism, and elimination [178]. This time-dependent aspect is especially important for anti-diabetic treatments since glucose tolerance, insulin sensitivity, and hepatic secretion of glucose all display well-characterized circadian rhythms [70]. Ignoring these rhythms could lead to inadequate outcomes, while dosing at a specific time could maximize drug activity.

An example of time-dependent pharmacological efficacy is metformin (first-line therapy in type 2 diabetes). Its primary mechanism, which is suppression of hepatic gluconeogenesis via AMPK activation, is tightly connected with circadian regulators such as BMAL1 and SIRT1 [179]. A study demonstrated that post-prandial administration during the active phase of the day (e.g., evening in diurnal humans) improves the action of metformin on fasting glucose levels and hepatic lipid metabolism [70], suggesting the use of metformin during active periods of the day. Likewise, sodium-glucose co-transporter 2 (SGLT2) inhibitors, which promote urinary glucose excretion, exhibit differences in efficacy and side effect profiles depending on the timing of administration. This variation is probably based on circadian control of renal glucose handling [180].

In addition to optimizing the timing of drug administration, chrono-pharmacology also offers a framework for targeting the circadian clock itself as a potential therapeutic target (Table 3). BMAL1 is a crucial component of the molecular clock, and its interaction with REV-ERBα serves a central function in the regulation of lipid metabolism, inflammation, and glucose homeostasis [174]. In preclinical models, synthetic agonists of REV-ERBs, such as SR9009, exert anti-obesity effects by decreasing adiposity, enhancing insulin sensitivity, and inhibiting hepatic lipogenesis [181]. Similarly, pharmacological activation of BMAL1 has been linked with improved mitochondrial function and protection against obesity caused by a HFD [182].

Basically, the activity of circadian targets is both tissue-specific and time-sensitive, reinforcing the necessity for clearly defined therapeutic windows. Thus, the orchestration of chronotherapy at a personalized scale must be accompanied by circumstantial introduction of circadian biomarkers, for example, through time-resolved transcriptomic or metabolomic profiles that could yield patient-specific treatment schedules for chronotype, lifestyle, and metabolic phenotype [183].

Chrono-pharmacology offers great potential from a clinical perspective in treating metabolic disorders in patient populations with specific disrupted circadian rhythms like night shift workers, elderly individuals, and patients with sleep disorders. However, despite promising preclinical data, larger randomized controlled trials are needed to establish the effectiveness and safety of time-restricted drug regimens and circadian-targeting therapeutics in populations with diverse backgrounds.

### 7.2. Circadian Nutritional Programming: The Role of Meal Timing in Redox Homeostasis

Chrono-nutrition—the study of how meal timing affects physiological processes—has emerged as a promising approach to optimizing metabolic health. Traditional dietary approaches to diet have focused on caloric intake or composition of macronutrients, whereas the concept of chrono-nutrition focuses on the timing of food intake relative to endogenous circadian rhythms [184]. Timing-based intervention gradually builds a great potential in restoring cellular redox homeostasis, optimal mitochondrial functioning, and regulation of glucose and lipid metabolism, which are severely compromised in various dysfunctional states of metabolism, such as obesity, insulin resistance, and MAFLD (Table 8).

Circadian rhythms are closely linked with cellular redox states. At the molecular level, NAD^+^/NADH ratios, mitochondrial dynamics, and ROS levels influence the activity of the key clock components like BMAL1, CLOCK, PER, and CRY [5]. Redox-sensitive transcription factors like Nrf2, in turn, orchestrate antioxidant defense pathways in a circadian manner, highlighting a bidirectional relationship between circadian regulation and redox homeostasis [6]. Such redox processes are tremendously affected by nutrient timing, especially because feeding can reset the peripheral clocks and modulate hepatic metabolic flux and OS responses, thus acting as a powerful zeitgeber (Table 8).

TRF—a dietary regimen in which food intake is confined to the organism’s active (awake) phase—has been shown to support the maintenance of circadian rhythms and redox homeostasis (Table 8). Notably, TRF confers protection against diet-induced obesity, insulin resistance, and hepatic steatosis in mice, even when total caloric intake is matched to that of ad libitum-fed controls [185]. TRF re-synchronizes the hepatic redox clock, enhancing the rhythmic expression of genes involved in mitochondrial biogenesis, antioxidant defense (e.g., *Nrf2*, *Sod2*), and fatty acid β-oxidation [173]. In contrast, food consumption during the rest phase misaligns circadian physiology, dampens metabolic rhythms, and increases oxidative damage. Additional studies are required to distinguish the effects of TRF from other intermittent fasting (IF) approaches, such as alternate-day fasting or 16:8 protocols, on hepatic circadian gene expression and redox control. Despite beneficial effects on insulin sensitivity and inflammation, the effects of these two strategies on clock-controlled redox enzymes and ROS-scavenging mechanisms can differ depending on the duration of fasting and its alignment with circadian timing [186]. Defining these differences will be essential for adapting chrono-nutritional strategies to metabolic disease-specific situations.

Another frontier in the field of chrono-nutrition research is the circadian regulation of the gut microbiota. The gut microbial community also displays oscillations in composition and metabolic activity over the day, reflecting feeding habits and the activity of host circadian genes [187]. Disruption of these rhythms, as seen in HFD or erratic eating schedules, promotes intestinal permeability, endotoxemia, and hepatic inflammation, all of them lead to OS. TRF has been reported to restore microbial rhythmicity, enrich beneficial taxa (e.g., Akkermansia), and augment microbial metabolites (e.g., short-chain fatty acids [SCFAs]) that maintain intestinal redox balance and promote hepatic mitochondrial function [188]. These results demonstrate the integrating role of chrono-nutrition in the modulation of both host metabolism and the gut-liver axis (Table 8).

In summary, meal timing is a potent zeitgeber that can coordinate peripheral clocks and regulate redox-sensitive pathways. By aligning food consumption with the circadian structure of the body, chrono-nutrition provides a mechanism for regulating antioxidant capacity, mitigating metabolic stress, and optimizing cardiometabolic outcomes. Future transcriptomic, metabolomic, and microbiome analyses will be crucial to designing personalized chrono-nutritional strategies for metabolic disease prevention and intervention.

## 8. Conclusions

Chrono-medicine is a rapidly evolving field that has the potential to transform our understanding of the cellular mechanisms that underlie metabolic and inflammatory disease pathogenesis. Herein, we highlight emerging conceptual frameworks that may address areas of knowledge within the field that have long required further elaboration.

First, they identify the circadian clock–proteasome–inflammasome axis as an important regulatory circuit linking time-dependent protein turnover, redox homeostasis, and innate immune activation, all of which are processes important in obesity, insulin resistance, and metabolic-associated steatohepatitis.

Second, circadian-sensitive endocrine and immune metabolic regulators have been identified in underexplored or neglected tissues like bone marrow adipose tissue and pink adipose tissue. These depots are involved in hematopoietic dynamics, thermogenesis, and systemic energy balance, making them relevant to circadian metabolic physiology.

Third, epigenetic circadian modulation, which includes rhythmic histone acetylation and chromatin remodeling, has recently emerged as a key driver of temporal regulation of metabolic gene expression and pathogenesis of metabolic-related diseases, such as metabolic dysfunction-associated fatty liver disease and liver fibrosis.

Together, these findings provide a rationale for using a systems biology perspective in circadian biology, one that integrates molecular, cellular, and tissue-specific oscillators with environmental signals and pathology phenotypes. As we move forward, a multidisciplinary approach will be essential. Translational chronotherapy, metabolomics, and precision medicine, coupled with molecular chronobiology, will likely contribute to the personalization of diagnostics and therapeutics based on the unique predominant circadian profiles of each individual.

Implementations of the principles of timed lifestyle interventions, including chrono-pharmacology, time-restricted nutrition, and targeted epigenomic modulation, have the potential to be game changers in the prevention and treatment of metabolic and inflammatory diseases, paving the way for precision medicine adapted to the dynamic nature of metabolism.

## Figures and Tables

**Table 1:** Glucocorticoid dysregulation and circadian metabolic imbalance.

Pathway/Process	Core Interaction	Effect on Systemic Physiology	References
Circadian regulation of GC secretion	GCs follow a circadian rhythm controlled by the SCN/PVN, regulating metabolism and immunity	Synchronizes peripheral clocks and regulates metabolism, immunity, and stress responses	[38]
BMAL1 and HPA axis control	BMAL1 modulates CRH, AVP, and REV-ERBα expression, linking circadian signals to GC secretion control	Ensures alignment between central clock and endocrine outputs	[39,40]
Chronic stress and Clock gene disruption	Chronic stress reprograms hepatic and systemic circadian rhythms, elevates fasting glucose, and dampens corticosterone rhythms	Disrupts glucose metabolism, immune regulation, and circadian gene expression	[41]
GR desensitization and metabolic dysfunction	Sustained GC exposure leads to GR downregulation and impaired circadian metabolic responses	Leads to insulin resistance, inflammation, and hepatic lipid accumulation	[42,43]
Tissue-specific GR activity in liver and adipose tissues	GCs regulate transcription and chromatin remodeling of metabolic genes (e.g., *Pck1*, *G6pc*, *Fasn*) in a tissue-specific manner	Coordinates metabolic adaptation in liver and adipose tissue; dysregulation contributes to disease	[44,45]
Chronotherapeutic targeting of GC rhythms	Selective GR modulators or timed inhibitors (e.g., metyrapone) restore rhythmic GC signaling and metabolic balance	Improves metabolic function and reduces effects of stress-induced circadian disruption	[42]
Lifestyle strategies to reinforce GC rhythms	Time-restricted feeding (TRF), light exposure, and structured activity reinforce endogenous GC oscillations	Boosts circadian alignment and enhances metabolic flexibility	[38]
Omics-guided chrono-endocrine precision medicine	Single-cell and time-series profiling help design personalized interventions based on GR sensitivity and circadian phase	Enables precision therapy for stress-related metabolic disorders like obesity and type 2 diabetes	[46]

**Table 2:** Circadian modulation of endocrine metabolism in adipose tissue.

Pathway/Process	Core Interaction	Effect on Systemic Physiology	References
Adipocyte intrinsic clocks and gene rhythms	BMAL1, CLOCK, PER, and CRY drive rhythmic expression of lipolytic, lipogenic, and adipokine genes in adipocytes	Coordinates adipose metabolism with daily cycles and feeding cues	[67,68]
Depot-specific circadian regulation	WAT, BAT, and visceral fat show distinct circadian expression patterns and responsiveness to environmental cues	Enables tailored metabolic and immune responses in different fat depots	[65,66]
Adipokine secretion rhythmicity	Leptin and adiponectin are secreted in a diurnal pattern, modulated by the circadian clock	Maintains energy balance and insulin sensitivity; disruption leads to leptin resistance	[69,70]
Circadian disruption and metabolic inflexibility	Disruption by shift work, stress, or irregular meals blunts hormonal rhythms, leading to metabolic dysfunction	Promotes obesity and type 2 diabetes through hormonal and immune imbalance	[71]
Circadian regulation of adipose-derived stem cells	ASC differentiation, mitochondrial activity, and inflammation follow circadian patterns influenced by zeitgebers	The timing of ASC use may improve regenerative and metabolic therapeutic outcomes	[71,72]
Sympathetic nervous system-adipose axis	SNS regulates diurnal lipolysis and thermogenesis; its disruption impairs metabolic flexibility	Modulates thermogenesis and lipolysis, critical for energy homeostasis	[73]
Chronotherapeutic potential in adipose tissue	Timing interventions such as feeding schedules or ASC transplantation may restore circadian adipose function	Supports the treatment of obesity and insulin resistance through circadian alignment	[68,74]

**Table 3:** Circadian control of proteostasis and redox balance.

Pathway/Process	Core Interaction	Effect on Metabolic Homeostasis	References
Proteasome–circadian interface	UPS activity shows circadian rhythmicity; CLOCK/BMAL1 regulate the expression of UPS components and protein turnover	Maintains temporal protein quality control in metabolically active tissues	[131,132,134,135]
BMAL1 and proteasome assembly	BMAL1 deficiency impairs proteasome assembly and degradation of oxidized/misfolded proteins	Loss of BMAL1 leads to proteotoxic stress and disrupted redox homeostasis	[24]
Circadian Control of Mitochondrial Proteostasis	Circadian degradation of PGC-1α and NRF1 coordinates mitophagy and mitochondrial biogenesis, linked to BMAL1	Promotes mitochondrial efficiency and minimizes ROS during metabolic transitions	[136,137]
Proteasomal disruption in metabolic disease	Obesity and hyperglycemia suppress proteasome rhythmicity, causing impaired protein turnover and redox imbalance	Dysregulated proteostasis contributes to inflammation and oxidative stress in obesity	[138,139]
Circadian Control of Antioxidant Defense	BMAL1 modulates *Nrf2* expression and degradation; links circadian rhythm to antioxidant gene regulation	Enhances antioxidant capacity and reduces inflammation through NRF2 stabilization	[35,140]

**Table 4:** Circadian inflammasome activation and metabolic inflammation.

Pathway/ Process	Core Interaction	Effect on Metabolic Homeostasis	References
Circadian regulation of inflammasomes	NLRP3 and AIM2 inflammasome activity shows circadian rhythmicity controlled by BMAL1, CLOCK, and REV-ERBα	Coordinates immune surveillance with daily metabolic cycles; its disruption leads to chronic low-grade inflammation	[9,144]
Oxidative stress and NLRP3 activation	ROS generated by mitochondrial dysfunction due to circadian disruption enhances inflammasome activation	Promotes inflammation in metabolically active tissues like the liver and adipose through increased ROS and inflammasome activity	[6,136,145]
Temporal misalignment and inflammation	Disrupted circadian rhythms (e.g., jet lag, HFD during rest phase) increase *NLRP3* expression and IL-1β production in liver and adipose tissues	Increases risk of insulin resistance, hepatic steatosis, and fibrosis by promoting immune cell infiltration	[146,147,148]
REV-ERBα and inflammasome inhibition	Pharmacological activation of REV-ERBα reduces NLRP3 activity and metabolic inflammation	Suppresses inflammasome-driven inflammation and improves metabolic outcomes in obesity models	[149]
BMAL1 and immune suppression	*BMAL1* deletion in myeloid cells enhances inflammasome priming and cytokine production	Limits innate immune activation and supports circadian control of immune homeostasis	[9]

**Table 5:** Circadian control of Bone Marrow and Pink Adipose tissue.

Pathway/Process	Core Interaction	Effect on Systemic Physiology	References
Circadian regulation of BMAT	BMAT expresses *Bmal1*, *Per2*, *Rev-erb alpha* with diurnal variation, suggesting the presence of an intrinsic clock	Links bone marrow metabolism to circadian rhythms, potentially affecting systemic energy balance	[145]
BMAT and hematopoietic crosstalk	BMAT may influence HSC maintenance and systemic inflammation through rhythmic secretion of lipids and adipokines	Coordinates metabolic and immune regulation within the bone marrow niche	[154,157]
BMAT exosomes and zeitgeber response	Exosomes from BMAT carry circadian-regulated miRNAs and proteins; their content is modulated by zeitgebers and stress	Disruption may promote inflammation, hematopoietic imbalance, and bone demineralization	[159]
BMAT and bone fragility	Circadian dysregulation of BMAT associated with impaired osteoblast differentiation and bone fragility	May contribute to osteoporosis pathogenesis under circadian disruption	[156,160]
Circadian role of pink adipose tissue	Pink adipose tissue emerges during lactation and may exhibit circadian plasticity in mitochondrial and secretory function	May play a role in thermogenic and secretory adaptation during specific physiological states	[161]

**Table 6:** Circadian epigenetic reprogramming in liver metabolic disorders.

Pathway/Process	Core Interaction	Effect on Systemic Physiology	References
Circadian histone acetylation in liver	Histone marks like H3K9ac and H3K4me3 are rhythmically modified and regulate hepatic gene expression	Synchronizes hepatic metabolism with feeding-fasting cycles; disruption promotes steatosis and inflammation	[164,165]
BMAL1 as a chromatin modifier	BMAL1 recruits p300 to enhancers, promoting circadian H3K9 acetylation at metabolic gene promoters	Coordinates transcription of genes involved in lipid metabolism and detoxification	[166,167]
Epigenetic disruption by HFD	HFD abolishes rhythmic histone modifications, disrupting transcriptional timing	Leads to liver susceptibility to MAFLD, inflammation, and fibrosis	[167,168]
DNA methylation and chromatin remodeling	Circadian disruption alters DNA methylation and chromatin structure, impairing hepatocyte function	Increases vulnerability to liver injury, OS, and functional decline	[169,170]
Chrono-epigenetics and therapeutic targeting	Chrono-pharmacology using HDAC inhibitors restores rhythmic gene expression and metabolism	Improves therapeutic outcomes in liver disease with time-optimized interventions	[167,171]
SIRT1 and time-dependent deacetylation	Targeting SIRT1 restores circadian chromatin dynamics and reduces hepatic steatosis	Enhances mitochondrial function and ameliorates metabolic liver disease	[89,172]
Behavioral restoration of circadian rhythms	TRF restores histone acetylation rhythmicity and protects against hepatic pathology	Entrains circadian rhythms independently of caloric restriction, protecting liver health	[173]

**Table 7:** Chrono-pharmacology and metabolic disease treatment.

Pathway/Process	Core Interaction	Effect on Systemic Physiology	References
Circadian control of drug metabolism	Circadian rhythms regulate drug absorption, distribution, metabolism, and elimination	Improves drug safety and therapeutic efficacy via optimized timing	[177,178]
Chrono-pharmacology principles	Drug effectiveness and side effects vary depending on the circadian phase of administration	Aligns pharmacodynamics with physiological rhythms, minimizing toxicity	[70,177]
Time-dependent metformin efficacy	Metformin action enhanced during active phase; linked to BMAL1 and SIRT1 rhythms	Improves glucose regulation and reduces hepatic lipid content	[70,179]
SGLT2 inhibitor chrono dynamics	Efficacy of SGLT2 inhibitors varies with dosing time, reflecting renal circadian control	Reduces adverse events and enhances glucose excretion	[180]
Targeting the molecular clock (BMAL1/REV-ERBα)	BMAL1 and REV-ERBα modulate lipid metabolism, inflammation, and glucose homeostasis	Synchronizes metabolic pathways for homeostasis	[174]
Pharmacological activation of circadian targets	Synthetic REV-ERB agonists reduce adiposity and improve insulin sensitivity; BMAL1 activation enhances mitochondrial function	Ameliorates obesity and supports energy metabolism	[181,182]
Personalized chronotherapy with biomarkers	Time-resolved transcriptomics and metabolomics guide treatment aligned to individual circadian profiles	Enables precision medicine for metabolic disorders	[183]
Clinical potential and chronotherapy trials	Potential for improved therapy in patients with circadian disruption; requires more Randomized Controlled Trialsfor validation	Targets metabolic dysregulation in vulnerable populations (e.g., shift workers)	[177,178]

**Table 8:** Circadian nutritional programming and redox homeostasis.

Pathway/Process	Core Interaction	Effect on Systemic Physiology	References
Chrono-nutrition and meal timing	Focuses on aligning eating patterns with circadian rhythms to optimize metabolic health	Improves glucose and lipid metabolism; supports redox balance in metabolic disease	[184]
Redox-circadian feedback loops	NAD^+^/NADH, ROS, and mitochondrial signals modulate clock gene expression and antioxidant defense	Coordinates mitochondrial function and antioxidant defenses	[5,6]
TRF	Restricting food intake to the active phase restores redox rhythms and circadian gene expression	Prevents diet-induced obesity, insulin resistance, and hepatic steatosis	[173,185]
TRF vs. intermittent fasting	Different fasting regimens vary in effect on redox enzymes and circadian alignment	TRF offers enhanced circadian benefits compared to other IF protocols	[186]
Gut microbiota rhythmicity	Microbial communities oscillate with feeding cycles and impact oxidative balance	Links circadian rhythms to gut health and inflammation control	[187]
TRF and gut-liver axis	TRF restores microbial oscillations, enhances SCFAs, and supports hepatic redox homeostasis	Modulates systemic inflammation and promotes mitochondrial efficiency	[188]
Nutrient timing and redox regulation	Feeding acts as a zeitgeber; proper timing enhances antioxidant capacity and metabolic resilience	Reduces metabolic stress and supports cardiometabolic health	[6,173]

## Abbreviations

The following abbreviations are used in this manuscript:

AIM2	Absent in melanoma 2
AMP	Adenosine monophosphate
AMPK	AMP-activated protein kinase
ARNT	Aryl hydrocarbon receptor nuclear translocator
AVPV	Hypothalamic anteroventral periventricular nucleus
ASC	Apoptosis-associated Speck-like protein containing a CARD
ATP	Adenosine triphosphate
BAT	Brown adipose tissue
BMAL1	Brain and muscle ARNT-like 1
BMAT	Bone marrow adipose tissue
BMI	Body mass index
CLOCK	Circadian locomotor output cycles kaput
GSK3β	Glycogen synthase kinase-3β
HDAC	Histone deacetylase
HFD	High-fat diet
HPG	Hypothalamic–pituitary–gonadal
HSC	Hematopoietic stem cell
hsCRP	High-sensitivity C-reactive protein
IL	Interleukin
LHPA	Lymbic hypothalamic–pituitary–adrenal
Kiss1	Kisspeptin gene in mammals
MAFLD	Metabolic dysfunction-associated fatty liver disease
MAsH	Metabolic dysfunction-associated steatohepatitis
MASL	Metabolic dysfunction-associated liver disease
MT2	Melatonin type-2
NF-κB	Nuclear factor kappa-light-chain-enhancer of activated B cells
NLRP3	NOD-like receptor family pyrin domain containing 3
NRF1	Nuclear respiratory factor 1
NRF2	Nuclear factor erythroid 2–related factor 2
PGC-1α	Peroxisome proliferator-activated receptor gamma coactivator 1-alpha
PKC	Protein kinase C
REV-ERBα	Nuclear receptor subfamily 1 group D member 1
ROS	Reactive oxygen species
SCN	Suprachiasmatic nucleus
TNF-α	Tumor necrosis factor-α
TRF	Time-restricted feeding
TXNIP	Thioredoxin-interacting protein
UPS	Ubiquitin–proteasome system
WAT	White adipose tissue

## References

[1] Partch C.L., Green C.B., Takahashi J.S. (2014). Molecular architecture of the mammalian circadian clock. Trends Cell Biol..

[2] Bass J., Takahashi J.S. (2010). Circadian integration of metabolism and energetics. Science.

[3] Opperhuizen A.-L., van Kerkhof L.W.M., Proper K.I., Rodenburg W., Kalsbeek A. (2015). Rodent models to study the metabolic effects of shiftwork in humans. Front. Pharmacol..

[4] Zimmet P., Alberti K.G.M.M., Stern N., Bilu C., El-Osta A., Einat H., Kronfeld-Schor N. (2019). The circadian syndrome: Is the metabolic syndrome and much more!. J. Intern. Med..

[5] Reinke H., Asher G. (2016). Circadian clock control of liver metabolic functions. Gastroenterology.

[6] Wilking M., Ndiaye M., Mukhtar H., Ahmad N. (2013). Circadian rhythm connections to oxidative stress: Implications for human health. Antioxid. Redox Signal..

[7] Pickel L., Sung H.-K. (2020). Feeding rhythms and the circadian regulation of metabolism. Front. Nutr..

[8] Zhang E.E., Liu A.C., Hirota T., Miraglia L.J., Welch G., Pongsawakul P.Y., Liu X., Atwood A., Huss J.W., Janes J. (2009). A genome-wide RNAi screen for modifiers of the circadian clock in human cells. Cell.

[9] Nguyen K.D., Fentress S.J., Qiu Y., Yun K., Cox J.S., Chawla A. (2013). Circadian gene Bmal1 regulates diurnal oscillations of Ly6C^hi^ inflammatory monocytes. Science.

[10] Betts J.A., Bowden Davies K.A., Smith H.A., Hawley J.A. Physiological rhythms and metabolic regulation: Shining light on skeletal muscle. Exp. Physiol..

[11] Procopio S.B., Esser K.A. (2025). Clockwork conditioning: Aligning the skeletal muscle clock with time-of-day exercise for cardiometabolic health. J. Mol. Cell. Cardiol..

[12] Stangherlin A., Seinkmane E., O’Neill J.S. (2021). Understanding circadian regulation of mammalian cell function, protein homeostasis, and metabolism. Curr. Opin. Syst. Biol..

[13] Bae K., Jin X., Maywood E.S., Hastings M.H., Reppert S.M., Weaver D.R. (2001). Differential functions of mPer1, mPer2, and mPer3 in the SCN circadian clock. Neuron.

[14] Liu A.C., Welsh D.K., Ko C.H., Tran H.G., Zhang E.E., Priest A.A., Buhr E.D., Singer O., Meeker K., Verma I.M. (2007). Intercellular coupling confers robustness against mutations in the SCN circadian clock network. Cell.

[15] Crown A., Lightman S. (2005). Management of patients with glucocorticoid deficiency. Nat. Clin. Pract. Endocrinol. Metab..

[16] Stratmann M., Schibler U. (2006). Properties, entrainment, and physiological functions of mammalian peripheral oscillators. J. Biol. Rhythms.

[17] McMahon D.G., Iuvone P.M., Tosini G. (2014). Circadian organization of the mammalian retina: From gene regulation to physiology and diseases. Prog. Retin. Eye Res..

[18] Pevet P., Challet E. (2011). Melatonin: Both Master Clock Output and Internal Time-Giver in the Circadian Clocks Network. J. Physiol. Paris.

[19] Zisapel N. (2018). New Perspectives on the Role of Melatonin in Human Sleep, Circadian Rhythms and Their Regulation. Br. J. Pharmacol..

[20] Dubocovich M.L., Rivera-Bermudez M.A., Gerdin M.J., Masana M.I. (2003). Molecular pharmacology, regulation and function of mammalian melatonin receptors. Front. Biosci..

[21] Hunt A.E., Al-Ghoul W.M., Gillette M.U., Dubocovich M.L. (2001). Activation of MT(2) melatonin receptors in rat suprachiasmatic nucleus phase advances the circadian clock. Am. J. Physiol. Cell Physiol..

[22] Pfeffer M., Rauch A., Korf H.W., von Gall C. (2012). The endogenous melatonin (MT) signal facilitates reentrainment of the circadian system to light-induced phase advances by acting upon MT2 receptors. Chronobiol. Int..

[23] Takahashi J.S. (2017). Transcriptional architecture of the mammalian circadian clock. Nat. Rev. Genet..

[24] Kondratov R.V., Kondratova A.A., Gorbacheva V.Y., Vykhovanets O.V., Antoch M.P. (2006). Early aging and age-related pathologies in mice deficient in BMAL1, the core component of the circadian clock. Genes Dev..

[25] Tomova A., Kumanov P., Robeva R., Manchev S., Konakchieva R. (2008). Melatonin secretion and non-specific immune responses are differentially expressed in corticotropin-dependent and corticotropin-independent Cushing’s syndrome. Med. Sci. Monit..

[26] Colombini B., Dinu M., Murgo E., Lotti S., Tarquini R., Sofi F., Mazzoccoli G. (2022). Ageing and low-level chronic inflammation: The role of the biological clock. Antioxidants.

[27] Cunningham P.S., Meijer P., Nazgiewicz A., Anderson S.G., Borthwick L.A., Bagnall J., Kitchen G.B., Lodyga M., Begley N., Venkateswaran R.V. (2020). The circadian clock protein REV-ERBα inhibits pulmonary fibrosis development. Proc. Natl. Acad. Sci. USA.

[28] Joshi A., Sundar I.K. (2023). Circadian disruption in night shift work and its association with chronic pulmonary diseases. Adv. Biol..

[29] Kiessling S., Teperino R. (2020). Circadian rhythms in health and disease. Beyond Our Genes.

[30] Lee J.H., Sancar A. (2011). Regulation of apoptosis by the circadian clock through NF-κB signaling. Proc. Natl. Acad. Sci. USA.

[31] Welz P.S., Benitah S.A. (2020). Molecular connections between circadian clocks and aging. J. Mol. Biol..

[32] Okamoto-Uchida Y., Izawa J., Nishimura A., Hattori A., Suzuki N., Hirayama J. (2019). Post-translational modifications are required for circadian clock regulation in vertebrates. Curr. Genom..

[33] Dang C.V. (2012). MYC on the Path to Cancer. Cell.

[34] Bozek K., Relógio A., Kielbasa S.M., Heine M., Dame C., Kramer A., Herzel H. (2009). Regulation of clock-controlled genes in mammals. PLoS ONE.

[35] Pekovic-Vaughan V., Gibbs J., Yoshitane H., Yang N., Pathiranage D., Guo B., Sagami A., Taguchi K., Bechtold D., Loudon A. (2014). The circadian clock regulates rhythmic activation of the NRF2/glutathione-mediated antioxidant defense pathway to modulate pulmonary fibrosis. Genes Dev..

[36] del Rey A., Chrousos G.P., Besedovsky H.O., Berczi I., Szentivanyi A. (2008). The Hypothalamus-Pituitary-Adrenal Axis. NeuroImmune Biology.

[37] Nicolaides N.C., Charmandari E., Chrousos G.P., Kino T. (2014). Circadian endocrine rhythms: The hypothalamic-pituitary-adrenal axis and its actions. Ann. N. Y. Acad. Sci..

[38] Matthews S.G., McGowan P.O. (2019). Developmental programming of the HPA axis and related behaviours: Epigenetic mechanisms. J. Endocrinol..

[39] Dickmeis T. (2009). Glucocorticoids and the circadian clock. J. Endocrinol..

[40] Lamia K.A., Papp S.J., Yu R.T., Barish G.D., Uhlenhaut N.H., Jonker J.W., Downes M., Evans R.M. (2011). Cryptochromes mediate rhythmic repression of the glucocorticoid receptor. Nature.

[41] Speksnijder E.M., Bisschop P.H., Siegelaar S.E., Stenvers D.J., Kalsbeek A. (2024). Circadian desynchrony and glucose metabolism. J. Pineal Res..

[42] Whirledge S., DeFranco D.B. (2018). Glucocorticoid signaling in heeremialth and disease: Insights from tissue-specific GR knockout mice. Endocrinology.

[43] Cohen S., Janicki-Deverts D., Doyle W.J., Miller G.E., Frank E., Rabin B.S., Turner R.B. (2012). Chronic stress, glucocorticoid receptor resistance, inflammation, and disease risk. Proc. Natl. Acad. Sci. USA.

[44] Præstholm S.M., Correia C.M., Grøntved L. (2020). Multifaceted control of GR signaling and its impact on hepatic transcriptional networks and metabolism. Front. Endocrinol..

[45] Man A.W.C., Xia N., Li H. (2020). Circadian rhythm in adipose tissue: Novel antioxidant target for metabolic and cardiovascular diseases. Antioxidants.

[46] Charmandari E., Tsigos C., Chrousos G. (2005). Endocrinology of the stress response. Annu. Rev. Physiol..

[47] Konakchieva R., Mitev Y., Almeida O.F.X., Patchev V. (1998). Chronic melatonin treatment counteracts glucocorticoid-induced dysregulation of the hypothalamic-pituitary-adrenal axis in the rat. Neuroendocrinology.

[48] Konakchieva R., Mitev Y., Almeida O.F.X., Patchev V. (1997). Chronic melatonin treatment and the HPA axis in the rat: Attenuation of the secretory response to stress and effects on hypothalamic neuropeptide content and release. Biol. Cell.

[49] Arendt J., Skene D.J. (2005). Melatonin as a chronobiotic. Sleep Med. Rev..

[50] Lund J., Arendt J., Hampton S.M., English J., Morgan L.M. (2001). Postprandial hormone and metabolic responses amongst shift workers in Antarctica. J. Endocrinol..

[51] Knutsson A. (2003). Health disorders of shift workers. Occup. Med..

[52] Karlsson H.K., Zierath J.R., Kane S., Krook A., Lienhard G.E., Wallberg-Henriksson H. (2005). Insulin-stimulated phosphorylation of the Akt substrate AS160 is impaired in skeletal muscle of type 2 diabetic subjects. Diabetes.

[53] Halberg F., Sothern R.B., Cornélissen G., Czaplicki J. (2008). Chronomics, human time estimation, and aging. Clin. Interv. Aging.

[54] Robeva R., Kirilov G., Tomova A., Kumanov P. (2008). Melatonin–insulin interactions in patients with metabolic syndrome. J. Pineal Res..

[55] Kondratova A.A., Kondratov R.V. (2012). The circadian clock and pathology of the ageing brain. Nat. Rev. Neurosci..

[56] Cinti S. (2019). White, brown, beige and pink: A rainbow in the adipose organ. Curr. Opin. Endocr. Metab. Res..

[57] Zorena K., Jachimowicz-Duda O., Ślęzak D., Robakowska M., Mrugacz M. (2020). Adipokines and obesity. Potential link to metabolic disorders and chronic complications. Int. J. Mol. Sci..

[58] Tratwal J., Rojas-Sutterlin S., Bataclan C., Blum S., Naveiras O. (2021). Bone marrow adiposity and the hematopoietic niche: A historical perspective of reciprocity, heterogeneity, and lineage commitment. Best Pract. Res. Clin. Endocrinol. Metab..

[59] Marinelli Busilacchi E., Morsia E., Poloni A. (2024). Bone Marrow Adipose Tissue. Cells.

[60] Kershaw E.E., Flier J.S. (2004). Adipose tissue as an endocrine organ. J. Clin. Endocrinol. Metab..

[61] Froy O., Garaulet M. (2018). The circadian clock in white and brown adipose tissue: Mechanistic, endocrine, and clinical aspects. Endocr. Rev..

[62] Fasshauer M., Bluher M. (2015). Adipokines in health and disease. Trends Pharmacol. Sci..

[63] Chouchani E.T., Kajimura S. (2019). Metabolic adaptation and maladaptation in adipose tissue. Nat. Metab..

[64] Emilova R., Dimitrova D.Z., Mladenov M., Hadzi-Petrushev N., Daneva T., Padeshki P., Schubert R., Chichova M., Lubomirov L., Simeonovska-Nikolova D. (2016). Diabetes converts arterial regulation by perivascular adipose tissue from relaxation into H_2_O_2_-mediated contraction. Physiol. Res..

[65] Kettner N.M., Mayo S.A., Hua J., Lee C., Moore D.D., Fu L. (2015). Circadian dysfunction induces leptin resistance in mice. Cell Metab..

[66] Gibbs J.E., Blaikley J., Beesley S., Matthews L., Simpson K.D., Boyce S.H., Farrow S.N., Else K.J., Singh D., Ray D.W. (2012). The nuclear receptor REV-ERBα mediates circadian regulation of innate immunity through selective regulation of inflammatory cytokines. Proc. Natl. Acad. Sci. USA.

[67] Zvonic S., Ptitsyn A.A., Conrad S.A., Scott L.K., Floyd Z.E., Kilroy G., Wu X., Goh B.C., Mynatt R.L., Gimble J.M. (2006). Characterization of peripheral circadian clocks in adipose tissues. Diabetes.

[68] Paschos G.K., Ibrahim S., Song W.L., Kunieda T., Grant G., Reyes T.M., Bradfield C.A., Vaughan C.H., Eiden M., Masoodi M. (2012). Obesity in mice with adipocyte-specific deletion of clock component Arntl. Nat. Med..

[69] Sáinz N., Barrenetxe J., Moreno-Aliaga M.J., Martínez J.A. (2015). Leptin resistance and diet-induced obesity: Central and peripheral actions of leptin. Metabolism.

[70] Qian J., Scheer F.A.J.L. (2016). Circadian system and glucose metabolism: Implications for physiology and disease. Trends Endocrinol. Metab..

[71] Perez L.M., Bernal A., de Lucas B., San Martin N., Mastrangelo A., García A., Sepúlveda P. (2018). Altered metabolic and stemness capacity of adipose tissue-derived stem cells from obese mouse and human. PLoS ONE.

[72] Ribas-Latre A., Bravo Santos R., Fekry B., Tamim Y.M., Shivshankar S., Mohamed A.M.T., Baumgartner C., Kwok C., Gebhardt C., Rivera A. (2021). Cellular and physiological circadian mechanisms drive diurnal cell proliferation and expansion of white adipose tissue. Nat. Commun..

[73] Cedernaes J., Waldeck N., Bass J. (2019). Neurogenetic basis for circadian regulation of metabolism by the hypothalamus. Genes Dev..

[74] Oster H., Challet E., Ott V., Arvat E., de Kloet E.R., Dijk D.-J., Lightman S., Vgontzas A., Van Cauter E. (2017). The functional and clinical significance of the 24-hour rhythm of circulating glucocorticoids. Endocr. Rev..

[75] Wu X., Zvonic S., Floyd Z.E., Kilroy G., Goh B.C., Hernandez T.L., Eckel R.H., Mynatt R.L., Gimble J.M. (2007). Induction of circadian gene expression in human subcutaneous adipose-derived stem cells. Obesity.

[76] Otway D.T., Mäntele S., Bretschneider S., Wright J., Trayhurn P., Skene D.J., Robertson M.D., Johnston J.D. (2011). Rhythmic diurnal gene expression in human adipose tissue from individuals who are lean, overweight, and type 2 diabetic. Diabetes.

[77] Civelek E., Ozturk Civelek D., Akyel Y.K., Kaleli Durman D., Okyar A. (2023). Circadian dysfunction in adipose tissue: Chronotherapy in metabolic diseases. Biology.

[78] Froy O. (2010). Metabolism and circadian rhythms—Implications for obesity. Endocr. Rev..

[79] Ramsey K.M., Marcheva B., Kohsaka A., Bass J. (2007). The clockwork of metabolism. Annu. Rev. Nutr..

[80] Albrecht U. (2017). The circadian clock, metabolism, and obesity. Obes. Rev..

[81] Barnea M., Madar Z., Froy O. (2010). High-fat diet followed by fasting disrupts circadian expression of adiponectin signaling pathway in muscle and adipose tissue. Obesity.

[82] Pivovarova O., Jürchott K., Rudovich N., Hornemann S., Ye L., Möckel S., Murahovschi V., Kessler K., Seltmann A.C., Maser-Gluth C. (2015). Changes of dietary fat and carbohydrate content alter central and peripheral clock in humans. J. Clin. Endocrinol. Metab..

[83] Preitner N., Damiola F., Lopez-Molina L., Zakany J., Duboule D., Albrecht U., Schibler U. (2002). The orphan nuclear receptor REV-ERBα controls circadian transcription within the positive limb of the mammalian circadian oscillator. Cell.

[84] Sato T.K., Panda S., Miraglia L.J., Reyes T.M., Rudic R.D., McNamara P., Naik K.A., FitzGerald G.A., Kay S.A., Hogenesch J.B. (2004). A functional genomics strategy reveals Rora as a component of the mammalian circadian clock. Neuron.

[85] Inoue I., Shinoda Y., Ikeda M., Hayashi K., Kanazawa K., Nomura M., Matsunaga T., Xu H., Kawai S., Awata T. (2005). CLOCK/BMAL1 is involved in lipid metabolism via transactivation of the peroxisome proliferator-activated receptor (PPAR) response element. J. Atheroscler. Thromb..

[86] Chen J., Xiang J., Zhou M., Huang R., Zhang J., Cui Y., Jiang X., Li Y., Zhou R., Xin H. (2025). Dietary timing enhances exercise by modulating fat-muscle crosstalk via adipocyte AMPKα2 signaling. Cell Metab..

[87] Mladenov M., Sazdova I., Hadzi-Petrushev N., Konakchieva R., Gagov H. (2025). The Role of Reductive Stress in the Pathogenesis of Endocrine-Related Metabolic Diseases and Cancer. Int. J. Mol. Sci..

[88] Sazdova I., Hadzi-Petrushev N., Keremidarska-Markova M., Stojchevski R., Sopi R., Shileiko S., Mitrokhin V., Gagov H., Avtanski D., Lubomirov L.T. (2024). SIRT-associated attenuation of cellular senescence in vascular wall. Mech. Ageing Dev..

[89] Keremidarska-Markova M., Sazdova I., Mladenov M., Pilicheva B., Zagorchev P., Gagov H. (2024). Sirtuin 1 and Hormonal Regulations in Aging. Appl. Sci..

[90] Ghesmati Z., Rashid M., Fayezi S., Gieseler F., Alizadeh E., Darabi M. (2024). An update on the secretory functions of brown, white, and beige adipose tissue: Towards therapeutic applications. Rev. Endocr. Metab. Disord..

[91] Shostak A., Meyer-Kovac J., Oster H. (2013). Circadian regulation of lipid mobilization in white adipose tissues. Diabetes.

[92] Unger R.H., Clark G.O., Scherer P.E., Orci L. (2010). Lipid homeostasis, lipotoxicity and the metabolic syndrome. Biochim. Biophys. Acta.

[93] Cano-Barquilla P., Jiménez-Ortega V., Fernández-Mateos P., Virto L., Maldonado Bautista E., Perez-Miguelsanz J., Esquifino A.I. (2025). Daily lipolysis gene expression in male rat mesenteric adipose tissue: Obesity and melatonin effects. Int. J. Mol. Sci..

[94] Kalsbeek A., Fliers E., Romijn J.A., La Fleur S.E., Wortel J., Bakker O., Endert E., Buijs R.M. (2001). The suprachiasmatic nucleus generates the diurnal changes in plasma leptin levels. Endocrinology.

[95] Kantermann T., Juda M., Merrow M., Roenneberg T. (2007). The human circadian clock’s seasonal adjustment is disrupted by daylight saving time. Curr. Biol..

[96] Fritz J., VoPham T., Wright K.P., Vetter C. (2020). A Chronobiological Evaluation of the Acute Effects of Daylight Saving Time on Traffic Accident Risk. Curr. Biol..

[97] Johnson K.G., Malow B.A. (2023). Implications of Sleep Health Policy: Daylight Saving and School Start Times. Continuum.

[98] Mladenov M., Lubomirov L., Grisk O., Avtanski D., Mitrokhin V., Sazdova I., Keremidarska-Markova M., Danailova Y., Nikolaev G., Konakchieva R. (2023). Oxidative Stress, Reductive Stress and Antioxidants in Vascular Pathogenesis and Aging. Antioxidants.

[99] Trujillo-Rangel W.Á., Acuña-Vaca S., Padilla-Ponce D.J., García-Mercado F.G., Torres-Mendoza B.M., Pacheco-Moises F.P., Escoto-Delgadillo M., García-Benavides L., Delgado-Lara D.L.C. (2024). Modulation of the Circadian Rhythm and Oxidative Stress as Molecular Targets to Improve Vascular Dementia: A Pharmacological Perspective. Int. J. Mol. Sci..

[100] Li X., Liu X., Meng Q., Wu X., Bing X., Guo N., Zhao X., Hou X., Wang B., Xia M. (2022). Circadian clock disruptions link oxidative stress and systemic inflammation to metabolic syndrome in obstructive sleep apnea patients. Front. Physiol..

[101] Deyurka N.A., Navigatore-Fonzo L.S., Coria-Lucero C.D., Ferramola M.L., Delgado S.M., Lacoste M.G., Anzulovich A.C. (2024). Aging abolishes circadian rhythms and disrupts temporal organization of antioxidant-prooxidant status, endogenous clock activity and neurotrophin gene expression in the rat temporal cortex. Neuroscience.

[102] Caturano A., D’Angelo M., Mormone A., Russo V., Mollica M.P., Salvatore T., Galiero R., Rinaldi L., Vetrano E., Marfella R. (2023). Oxidative Stress in Type 2 Diabetes: Impacts from Pathogenesis to Lifestyle Modifications. Curr. Issues Mol. Biol..

[103] Furukawa S., Fujita T., Shimabukuro M., Iwaki M., Yamada Y., Nakajima Y., Nakayama O., Makishima M., Matsuda M., Shimomura I. (2004). Increased oxidative stress in obesity and its impact on metabolic syndrome. J. Clin. Investig..

[104] Rajkovic N., Zamaklar M., Lalic K., Jotic A., Lukic L., Milicic T., Singh S., Stosic L., Lalic N.M. (2014). Relationship between Obesity, Adipocytokines and Inflammatory Markers in Type 2 Diabetes: Relevance for Cardiovascular Risk Prevention. Int. J. Environ. Res. Public Health.

[105] Budkowska M., Cecerska-Heryć E., Marcinowska Z., Siennicka A., Dołęgowska B. (2022). The Influence of Circadian Rhythm on the Activity of Oxidative Stress Enzymes. Int. J. Mol. Sci..

[106] Xia N., Li H.G. (2017). The role of perivascular adipose tissue in obesity-induced vascular dysfunction. Br. J. Pharmacol..

[107] Fuster J.J., Ouchi N., Gokce N., Walsh K. (2016). Obesity-Induced Changes in Adipose Tissue Microenvironment and Their Impact on Cardiovascular Disease. Circ. Res..

[108] Cildir G., Akıncılar S.C., Tergaonkar V. (2013). Chronic adipose tissue inflammation: All immune cells on the stage. Trends Mol. Med..

[109] Robertson J.L., Clifton D.K., de la Iglesia H.O., Steiner R.A., Kauffman A.S. (2009). Circadian regulation of Kiss1 neurons: Implications for timing the preovulatory gonadotropin-releasing hormone/luteinizing hormone surge. Endocrinology.

[110] Ono M., Ando H., Daikoku T., Fujiwara T., Mieda M., Mizumoto Y., Iizuka T., Kagami K., Hosono T., Nomura S. (2023). The Circadian Clock, Nutritional Signals and Reproduction: A Close Relationship. Int. J. Mol. Sci..

[111] Padda J., Khalid K., Moosa A., Syam M., Kakani V., Imdad U., Ismail D., Cooper A.C., Jean-Charles G. (2021). Role of Kisspeptin on Hypothalamic-Pituitary-Gonadal Pathology and Its Effect on Reproduction. Cureus.

[112] Xie Q., Kang Y., Zhang C., Xie Y., Wang C., Liu J., Yu C., Zhao H., Huang D. (2022). The role of kisspeptin in the control of the hypothalamic-pituitary-gonadal axis and reproduction. Front. Endocrinol..

[113] Neal-Perry G., Lebesgue D., Lederman M., Shu J., Zeevalk G.D., Etgen A.M. (2009). The excitatory peptide kisspeptin restores the luteinizing hormone surge and modulates amino acid neurotransmission in the medial preoptic area of middle-aged rats. Endocrinology.

[114] Jayasena C.N., Nijher G.M., Comninos A.N., Abbara A., Januszewki A., Vaal M.L., Sriskandarajah L., Murphy K.G., Farzad Z., Ghatei M.A. (2011). The effects of kisspeptin-10 on reproductive hormone release show sexual dimorphism in humans. J. Clin. Endocrinol. Metab..

[115] Chassard D., Bur I., Poirel V.J., Mendoza J., Simonneaux V. (2015). Evidence for a Putative Circadian Kiss-Clock in the Hypothalamic AVPV in Female Mice. Endocrinology.

[116] Smarr B.L., Morris E., de la Iglesia H.O. (2012). The dorsomedial suprachiasmatic nucleus times circadian expression of Kiss1 and the luteinizing hormone surge. Endocrinology.

[117] Padilla S.L., Perez J.G., Ben-Hamo M., Johnson C.W., Sanchez R.E.A., Bussi I.L., Palmiter R.D., de la Iglesia H.O. (2019). Kisspeptin neurons in the arcuate nucleus of the hypothalamus orchestrate circadian rhythms and metabolism. Curr. Biol..

[118] Brown R.E., Imran S.A., Ur E., Wilkinson M. (2008). KiSS-1 mRNA in adipose tissue is regulated by sex hormones and food intake. Mol. Cell. Endocrinol..

[119] Hussain M.A., Song W.J., Wolfe A. (2015). There is Kisspeptin—And Then There is Kisspeptin. Trends Endocrinol. Metab..

[120] Song W.J., Mondal P., Wolfe A., Alonso L.C., Stamateris R., Ong B.W., Lim O.C., Yang K.S., Radovick S., Novaira H.J. (2014). Glucagon regulates hepatic kisspeptin to impair insulin secretion. Cell Metab..

[121] Tolson K.P., Garcia C., Yen S., Simonds S., Stefanidis A., Lawrence A., Smith J.T., Kauffman A.S. (2014). Impaired kisspeptin signaling decreases metabolism and promotes glucose intolerance and obesity. J. Clin. Investig..

[122] Wahab F., Bano R., Jabeen S., Irfan S., Shahab M. (2010). Effect of peripheral kisspeptin administration on adiponectin, leptin, and resistin secretion under fed and fasting conditions in the adult male rhesus monkey (*Macaca mulatta*). Horm. Metab. Res..

[123] Khoramipour K., Chamari K., Hekmatikar A.A., Ziyaiyan A., Taherkhani S., Elguindy N.M., Bragazzi N.L. (2021). Adiponectin: Structure, Physiological Functions, Role in Diseases, and Effects of Nutrition. Nutrients.

[124] Pruszyńska-Oszmałek E., Kołodziejski P., Sassek M., Sliwowska J.H. (2017). Kisspeptin-10 inhibits proliferation and regulates lipolysis and lipogenesis processes in 3T3-L1 cells and isolated rat adipocytes. Endocrine.

[125] Yeo S.H., Colledge W.H. (2018). The Role of Kiss1 Neurons As Integrators of Endocrine, Metabolic, and Environmental Factors in the Hypothalamic-Pituitary-Gonadal Axis. Front. Endocrinol..

[126] Navarro V.M. (2020). Metabolic regulation of kisspeptin—The link between energy balance and reproduction. Nat. Rev. Endocrinol..

[127] Rivera H.M., Stincic T.L. (2018). Estradiol and the control of feeding behavior. Steroids.

[128] Allan C.A., McLachlan R.I. (2010). Androgens and obesity. Curr. Opin. Endocrinol. Diabetes Obes..

[129] Asarian L., Geary N. (2006). Modulation of appetite by gonadal steroid hormones. Philos. Trans. R. Soc. Lond. B Biol. Sci..

[130] Forbes S., Li X.F., Kinsey-Jones J., O’Byrne K. (2009). Effects of ghrelin on Kisspeptin mRNA expression in the hypothalamic medial preoptic area and pulsatile luteinising hormone secretion in the female rat. Neurosci. Lett..

[131] Zhao L., Zhao J., Zhong K., Tong A., Jia D. (2022). Targeted protein degradation: Mechanisms, strategies and application. Signal Transduct. Target. Ther..

[132] Desvergne A., Ugarte N., Radjei S., Gareil M., Petropoulos I., Friguet B. (2016). Circadian modulation of proteasome activity and accumulation of oxidized protein in human embryonic kidney HEK 293 cells and primary dermal fibroblasts. Free Radic. Biol. Med..

[133] Shi S.-Q., Ansari T.S., McGuinness O.P., Wasserman D.H., Johnson C.H. (2013). Circadian disruption leads to insulin resistance and obesity. Curr. Biol..

[134] Adamovich Y., Rousso-Noori L., Zwighaft Z., Neufeld-Cohen A., Golik M., Kraut-Cohen J., Wang M., Han X., Asher G. (2014). Circadian clocks and feeding time regulate the oscillations and levels of hepatic triglycerides. Cell Metab..

[135] Robles M.S., Humphrey S.J., Mann M. (2017). Phosphorylation is a central mechanism for circadian control of metabolism and physiology. Cell Metab..

[136] Lee J., Moulik M., Fang Z., Saha P., Zou F., Xu Y., Nelson D.L., Ma K., Moore D.D., Yechoor V.K. (2013). Bmal1 and β-cell clock are required for adaptation to circadian disruption, and their loss of function leads to oxidative stress-induced β-cell failure in mice. Mol. Cell. Biol..

[137] Mezhnina V., Ebeigbe O.P., Poe A., Kondratov R.V. (2022). Circadian control of mitochondria in reactive oxygen species homeostasis. Antioxid. Redox Signal..

[138] Su K.-H., Dai C. (2016). Metabolic control of the proteotoxic stress response: Implications in diabetes mellitus and neurodegenerative disorders. Cell. Mol. Life Sci..

[139] Bartelt A., Lemmer I., Willemsen N., Kotschi S., Toksöz I., Gjika E., Khani S., Haas D., Krahmer N., Rohm M. Nfe2l1-Mediated Proteasome Function Controls Muscle Energy Metabolism in Obesity. Res. Sq..

[140] Early J.O., Menon D., Wyse C.A., Cervantes-Silva M.P., Zaslona Z., Carroll R.G., Palsson-McDermott E.M., Angiari S., Ryan D.G., Corcoran S.E. (2018). Circadian Clock Protein BMAL1 Regulates IL-1β in Macrophages via NRF2. Proc. Natl. Acad. Sci. USA.

[141] Proietti S., Cucina A., Dobrowolny G., D’Anselmi F., Dinicola S., Masiello M.G., Pasqualato A., Palombo A., Morini V., Reiter R.J. (2014). Melatonin Down-Regulates MDM2 Gene Expression and Enhances P53 Acetylation in MCF-7 Cells. J. Pineal Res..

[142] Hill S.M., Frasch T., Xiang S., Yuan L., Duplessis T., Mao L. (2009). Molecular Mechanisms of Melatonin Anticancer Effects. Integr. Cancer Ther..

[143] Guo H., Callaway J.B., Ting J.P.-Y. (2015). Inflammasomes: Mechanism of Action, Role in Disease, and Therapeutics. Nat. Med..

[144] Silver A.C., Arjona A., Walker W.E., Fikrig E. (2012). The Circadian Clock Controls Toll-Like Receptor 9-Mediated Innate and Adaptive Immunity. Immunity.

[145] Zhang R., Lahens N.F., Ballance H.I., Hughes M.E., Hogenesch J.B. (2014). A Circadian Gene Expression Atlas in Mammals: Implications for Biology and Medicine. Proc. Natl. Acad. Sci. USA.

[146] Marcheva B., Ramsey K.M., Buhr E.D., Kobayashi Y., Su H., Ko C.H., Ivanova G., Omura C., Mo S., Vitaterna M.H. (2010). Disruption of the Clock Components CLOCK and BMAL1 Leads to Hypoinsulinaemia and Diabetes. Nature.

[147] Chassaing B., Etienne-Mesmin L., Gewirtz A.T. (2014). Microbiota–Liver Axis in Hepatic Disease. Hepatology.

[148] Hotamisligil G.S. (2017). Inflammation, Metaflammation and Immunometabolic Disorders. Nature.

[149] Pourcet B., Zecchin M., Ferri L., Beauchamp J., Sitaula S., Billon C., Delhaye S., Vanhoutte J., Mayeuf-Louchart A., Thorel Q. (2018). NR1D1 Regulates Circadian Activity of NLRP3 Inflammasome to Reduce Severity of Fulminant Hepatitis in Mice. Gastroenterology.

[150] Arioz B.I., Tarakcioglu E., Olcum M., Genc S. (2021). The Role of Melatonin on NLRP3 Inflammasome Activation in Diseases. Antioxidants.

[151] Garcia J.A., Volt H., Venegas C., Doerrier C., Escames G., Lopez L.C., Acuna-Castroviejo D. (2015). Disruption of the NF-κB/NLRP3 Connection by Melatonin Requires RORα and Blocks the Septic Response in Mice. FASEB J..

[152] Cao Z., Fang Y., Lu Y., Tan D., Du C., Li Y., Ma Q., Yu J., Chen M., Zhou C. (2017). Melatonin Alleviates Cadmium-Induced Liver Injury by Inhibiting the TXNIP-NLRP3 Inflammasome. J. Pineal Res..

[153] Xu B., Jiang M., Chu Y., Wang W., Chen D., Li X., Zhang Z., Zhang D., Fan D., Nie Y. (2018). Gasdermin D Plays a Key Role as a Pyroptosis Executor of Non-Alcoholic Steatohepatitis in Humans and Mice. J. Hepatol..

[154] Scheller E.L., Khandaker S., Learman B.S., Cawthorn W.P., Anderson L.M., Pham H.A., Robles H., Wang Z., Li Z., Parlee S.D. (2019). Bone Marrow Adipocytes Resist Lipolysis and Remodeling in Response to β-Adrenergic Stimulation. Bone.

[155] Cawthorn W.P., Scheller E.L., Parlee S.D., Pham H.A., Learman B.S., Redshaw C.M.H., Sulston R.J., Burr A.A., Das A.K., Simon B.R. (2016). Expansion of Bone Marrow Adipose Tissue During Caloric Restriction Is Associated with Increased Circulating Glucocorticoids. Endocrinology.

[156] Pachón-Peña G., Bredella M.A. (2022). Bone Marrow Adipose Tissue in Metabolic Health. Trends Endocrinol. Metab..

[157] Casanova-Acebes M., Pitaval C., Weiss L.A., Nombela-Arrieta C., Chèvre R., A-González N., Kunisaki Y., Zhang D., van Rooijen N., Silberstein L.E. (2013). Rhythmic Modulation of the Hematopoietic Niche through Neutrophil Clearance. Cell.

[158] Khalyfa A., Gaddameedhi S., Crooks E., Zhang C., Li Y., Qiao Z., Trzepizur W., Kay S.A., Andrade J., Satterfield B.C. (2020). Circulating Exosomal miRNAs Signal Circadian Misalignment to Peripheral Metabolic Tissues. Int. J. Mol. Sci..

[159] Tencerova M., Kassem M. (2016). The Bone Marrow-Derived Stromal Cells: Commitment and Regulation of Adipogenesis. Front. Endocrinol..

[160] McGrath C., Sankaran J.S., Misaghian-Xanthos N., Sen B., Xie Z., Styner M.A., Zong X., Rubin J., Styner M. (2020). Exercise Degrades Bone in Caloric Restriction, Despite Suppression of Marrow Adipose Tissue (MAT). J. Bone Miner. Res..

[161] Cinti S. (2012). The Adipose Organ at a Glance. Dis. Model. Mech..

[162] Hardouin P., Rharass T., Lucas S. (2016). Bone Marrow Adipose Tissue: To Be or Not to Be a Typical Adipose Tissue?. Front. Endocrinol..

[163] Feng D., Liu T., Sun Z., Bugge A., Mullican S.E., Alenghat T., Liu X.S., Lazar M.A. (2011). A Circadian Rhythm Orchestrated by Histone Deacetylase 3 Controls Hepatic Lipid Metabolism. Science.

[164] Koike N., Yoo S.-H., Huang H.-C., Kumar V., Lee C., Kim T.-K., Takahashi J.S. (2012). Transcriptional architecture and chromatin landscape of the core circadian clock in mammals. Science.

[165] Morral N., Liu S., Conteh A.M., Chu X., Wang Y., Dong X.C., Liu Y., Linnemann A.K., Wan J. (2021). Aberrant gene expression induced by a high-fat diet is linked to H3K9 acetylation in the promoter-proximal region. Biochim. Biophys. Acta Gene Regul. Mech..

[166] Hirayama J., Sahar S., Grimaldi B., Tamaru T., Takamatsu K., Nakahata Y., Sassone-Corsi P. (2007). CLOCK-mediated acetylation of BMAL1 controls circadian function. Nature.

[167] Masri S., Rigor P., Cervantes M., Ceglia N., Sebastian C., Xiao C., Roqueta-Rivera M., Deng C., Osborne T.F., Mostoslavsky R. (2014). Partitioning circadian transcription by SIRT6 leads to segregated control of cellular metabolism. Cell.

[168] Ding L., Liu J., Zhou L., Jia X., Li S., Zhang Q., Yu M., Xiao X. (2022). A high-fat diet disrupts the hepatic and adipose circadian rhythms and modulates the diurnal rhythm of gut microbiota-derived short-chain fatty acids in gestational mice. Front. Nutr..

[169] Asif S., Morrow N.M., Mulvihill E.E., Kim K.-H. (2020). Understanding dietary intervention-mediated epigenetic modifications in metabolic diseases. Front. Genet..

[170] Maude H., Sanchez-Cabanillas C., Cebola I. (2021). Epigenetics of hepatic insulin resistance. Front. Endocrinol..

[171] Woller A., Duez H., Staels B., Lefranc M. (2016). A mathematical model of the liver circadian clock linking feeding and fasting cycles to clock function. Cell Rep..

[172] Chang H.-C., Guarente L. (2014). SIRT1 and other sirtuins in metabolism. Trends Endocrinol. Metab..

[173] Chaix A., Zarrinpar A., Miu P., Panda S. (2014). Time-restricted feeding is a preventative and therapeutic intervention against diverse nutritional challenges. Cell Metab..

[174] Zhang Y., Fang B., Emmett M.J., Damle M., Sun Z., Feng D., Armour S.M., Remsberg J.R., Jager J., Soccio R.E. (2015). Discrete functions of nuclear receptor REV-ERBα couple metabolism to the clock. Science.

[175] Eckel-Mahan K.L., Patel V.R., de Mateo S., Orozco-Solis R., Ceglia N.J., Sahar S., Dilag-Penilla S.A., Dyar K.A., Baldi P., Sassone-Corsi P. (2013). Reprogramming of the circadian clock by nutritional challenge. Cell.

[176] Masri S., Patel V.R., Eckel-Mahan K.L., Peleg S., Forne I., Ladurner A.G., Baldi P., Imhof A., Sassone-Corsi P. (2013). Circadian acetylome reveals regulation of mitochondrial metabolic pathways. Proc. Natl. Acad. Sci. USA.

[177] Bass J., Lazar M.A. (2016). Circadian time signatures of fitness and disease. Science.

[178] Hermida R.C., Ayala D.E., Smolensky M.H., Fernández J.R., Mojón A., Portaluppi F. (2016). Chronotherapy with conventional blood pressure medications improves management of hypertension and reduces cardiovascular and stroke risks. Hypertens. Res..

[179] Um J.-H., Park S.-J., Kang H., Yang S., Foretz M., McBurney M.W., Kim M.K., Viollet B., Chung J.H. (2010). AMP-activated protein kinase-deficient mice are resistant to the metabolic effects of resveratrol. Diabetes.

[180] Reddy A.B., O’Neill J.S. (2010). Healthy clocks, healthy body, healthy mind. Trends Cell Biol..

[181] Solt L.A., Wang Y., Banerjee S., Hughes T., Kojetin D.J., Lundasen T., Shin Y., Liu J., Cameron M.D., Noel R. (2012). Regulation of circadian behavior and metabolism by synthetic REV-ERB agonists. Nature.

[182] Wang X.-L., Kooijman S., Gao Y., Tzeplaeff L., Cosquer B., Milanova I., Wolff S.E.C., Korpel N., Champy M.-F., Petit-Demoulière B. (2021). Microglia-specific knock-down of *Bmal1* improves memory and protects mice from high-fat diet-induced obesity. Mol. Psychiatry.

[183] Skarke C., Lahens N.F., Rhoades S.D., Campbell A., Bittinger K., Bailey A., Hoffmann C., Olson R.S., Chen L., Yang G. (2017). A pilot characterization of the human chronobiome. Sci. Rep..

[184] Almoosawi S., Prynne C.J., Hardy R., Stephen A.M. (2013). Time-of-day and nutrient composition of eating occasions: Prospective association with the metabolic syndrome in the 1946 British birth cohort. Int. J. Obes..

[185] Hatori M., Vollmers C., Zarrinpar A., DiTacchio L., Bushong E.A., Gill S., Leblanc M., Chaix A., Joens M., Fitzpatrick J.A.J. (2012). Time-restricted feeding without reducing caloric intake prevents metabolic diseases in mice fed a high-fat diet. Cell Metab..

[186] Longo V.D., Panda S. (2016). Fasting, circadian rhythms, and time-restricted feeding in healthy lifespan. Cell Metab..

[187] Thaiss C.A., Zeevi D., Levy M., Zilberman-Schapira G., Suez J., Tengeler A.C., Abramson L., Katz M.N., Korem T., Zmora N. (2014). Transkingdom control of microbiota diurnal oscillations promotes metabolic homeostasis. Cell.

[188] Liang X., Bushman F.D., FitzGerald G.A. (2015). Rhythmicity of the intestinal microbiota is regulated by gender and the host circadian clock. Proc. Natl. Acad. Sci. USA.

